# The family of glutathione peroxidase proteins and their role against biotic stress in plants: a systematic review

**DOI:** 10.3389/fpls.2025.1425880

**Published:** 2025-02-20

**Authors:** Maria Luíza do Carmo Santos, Ariana Silva Santos, Diogo Pereira Silva de Novais, Natasha dos Santos Lopes, Carlos Priminho Pirovani, Fabienne Micheli

**Affiliations:** ^1^ Universidade Estadual de Santa Cruz (UESC), Departamento de Ciências Biológicas (DCB), Centro de Biotecnologia e Genética (CBG), Ilhéus, Brazil; ^2^ Instituto Federal de Educação Ciência e Tecnologia da Bahia (IFBA), Bahia, Brazil; ^3^ CIRAD, UMR AGAP, Montpellier, France

**Keywords:** antioxidant action, defense responses, pathogens, cell death, plant disease

## Abstract

**Introduction:**

Glutathione peroxidases (GPXs) are extensively studied for their indispensable roles in eliminating reactive oxygen species by catalyzing the reduction of hydrogen peroxide or lipid peroxides to prevent cell damage. However, knowledge of GPXs in plants still has many gaps to be filled. Thus, we present the first systematic review (SR) aimed at examining the function of GPXs and their protective role against cell death in plants subjected to biotic stress.

**Methods:**

To guide the SR and avoid bias, a protocol was developed that contained inclusion and exclusion criteria based on PRISMA guidelines. Three databases (PubMed, Science Direct, and Springer) were used to identify relevant studies for this research were selected.

**Results:**

A total of 28 articles related to the proposed objective. The results highlight the importance of GPXs in plant defense against biotic stress, including their role in protecting against cell death, similar to the anti-apoptotic GPXs in animals. Data from gene expression and protein accumulation studies in plants under various biotic stresses reveal that GPXs can both increase resistance and susceptibility to pathogens. In addition to their antioxidant functions, GPXs act as sensors and transmitters of H_2_O_2_ signals, integrating with the ABA signaling pathway during stress.

**Discussion:**

These findings show that GPXs delay senescence or reinforce physical barriers, thereby modulating resistance or susceptibility to pathogens. Additionally, their functions are linked to their cellular localization, which demonstrates an evolutionary relationship between the studied isoforms and their role in plant defense. This information broadens the understanding of molecular strategies involving GPX isoforms and provides a foundation for discussions and actions aimed at controlling necrotrophic and/or hemibiotrophic pathogens.

## 1 Introduction

Plants are constantly exposed to various stress conditions. Among these, biotic stresses involve significant losses in agricultural yield and food production, affecting the global economy (Pereira, 1989; Blomme et al., 2017; Moreira et al., 2022; Ofori et al., 2022). These problems are caused by various phytopathogenic organisms, such as microorganisms and insects, each with different infection, development, and survival strategies within the host (Meng et al., 2009; Vojnov et al., 2010; Doehlemann et al., 2017; Noman et al., 2021). In most of the responses to stress, the plant promotes an increase in reactive oxygen species (ROS) (Dias et al., 2011; Mittler et al., 2022) that can cause damage such as lipid degradation, protein degradation, nucleic acid damage, and induce programmed cell death (PCD) (Das and Roychoudhury, 2014).

However, plants possess an antioxidant system that protects against damage caused by ROS, composed of enzymatic and non-enzymatic elements (Dumanović et al., 2020). Among the enzymatic components, glutathione peroxidases (GPXs) play a crucial role in resistance to stresses induced by phytopathogens (Rockenbach et al., 2015; Wang et al., 2018). These proteins catalyze the conversion of hydrogen peroxide (H_2_O_2_) and organic or lipid hydroperoxides into water or alcohols, using glutathione (GSH) or thioredoxin (Trx) as electron donors (Arthur, 2000; Iqbal et al., 2006; Bela et al., 2022). In addition, they contribute to the regulation of cellular redox homeostasis, maintaining the thiol/disulfide and NADPH/NADP+ balance (Bela et al., 2015).

Generally, proteins of the GPX family are characterized either as selenoproteins or non-selenoproteins depending if they have in their catalytic sites the amino acid residue selenocysteine (SeCys) encoded by the UGA codon or the cysteine (Cys) encoded by the UGU or UGC codon (Trenz et al., 2021). GPXs have three conserved motifs, which although poorly detailed regarding their function, are present in most eukaryotic organisms. In plants, these motifs are VNAS[R/K/Q]CG, LAFPCNQF, and WNF(S/T)KF, wherein three primary catalytic centers are presented as cysteine (Cys) or selenocysteine (Cys)-Glutamine (Gln)-Tryptophan (Trp) (Churin et al., 1999). However, molecular biology techniques of targeted mutations using CRISPR/Cas, as well as functional studies of protein production and characterization, with activity assays, in addition to *in silico* and *in vitro* studies of protein-ligand or protein-protein interactions, could clarify the specific roles of these motifs (Létoffé et al., 1998; Scheerer et al., 2007; Shi et al., 2010; Martins Alves et al., 2019; Dolgalev and Poverennaya, 2021; Avery et al., 2022). Furthermore, plant GPXs vary in terms of cellular localization (e.g. chloroplast, mitochondria, cytoplasm, or apoplast) (Navrot et al., 2006; Yang et al., 2006; Attacha et al., 2017), and their function are still poorly understood and explored. The cellular localization of these enzymes is crucial for their protective functions against biotic stress, as it is linked to their effectiveness in providing protection (Mbemba et al., 1985; Navrot et al., 2006). In the chloroplast, during pathogen infections, ROS production can increase significantly, and GPX helps mitigate this stress by protecting photosynthetic components and potentially reducing PCD (Mbemba et al., 1985; Yoshimura et al., 2004; Zhai et al., 2013). Mitochondria, similarly, play a crucial role in preventing oxidative damage to respiratory components. In peroxisomes, where antioxidant enzymes are commonly present, GPXs also help manage H_2_O_2_ levels during biotic stress (Rodríguez-Serrano et al., 2016). The apoplast, a critical site for host-pathogen interactions, benefits from extracellular or apoplastic GPXs that reduce ROS levels, thereby helping the plant prevent tissue damage (Foyer and Noctor, 2005). In the nucleus, GPXs protect DNA from oxidative damage and are involved in regulating gene expression during stress responses (Gaber et al., 2012). The localization of these plant enzymes can be investigated using techniques such as localized gene expression studies, cell fractionation, Western blot analysis, immunolocalization with electron microscopy, fusion with fluorescent proteins, as well as transcriptomics and proteomics of cellular compartments (Parish, 1971; Herbette et al., 2004; Zhai et al., 2013; de Paula and Techio, 2014; Chen et al., 2017; Arnaud et al., 2022; Christopher et al., 2022; Wang et al., 2023).

Systematizing published data on GPXs is crucial for understanding their functions and role in mitigating damage induced by pathogens, including cell death, which is vital for advancing future research and potential applications of GPXs in plant responses to biotic stress. Employing a Systematic Review (SR) approach ensures a comprehensive and unbiased compilation of high-quality investigations, akin to its common utilization in the medical field (Morvaridzadeh et al., 2021; Wang et al., 2021; Zhao et al., 2021; Pereira et al., 2022). While traditionally applied in medicine, recent years have seen an increasing exploration of SRs in agronomic studies, particularly in elucidating plant-pathogen interaction mechanisms encompassing molecular, biological, and biochemical responses (Soares et al., 2021; de Novais et al., 2023; dos Santos Lopes et al., 2023; Santos et al., 2023). In this context, our study represents the first SR on plant GPXs concerning biotic stress, revealing their antioxidant activity and diverse functions, including the potential inhibition of cell death, similarly findings in animal systems. This work sets the stage for further research into GPXs, paving the way for hypothesis validations and the discovery of new functionalities crucial for enhancing plant resilience to biotic stresses.

## 2 Methods

### 2.1 Systematic review protocol

The SR was conducted using R version 4.0.3 (R.C. Team, 2023) with the Bibliometrix package and the StArt (State of the Art through Systematic Review) software version Beta 3.0.3 (Fabbri et al., 2016). This software was developed by the Federal University of São Carlos (UFSCar) and is available for download (http://lapes.dc.ufscar.br/tools/start_tool). The review followed the PRISMA (Preferred Reporting Items for Systematic Reviews and Meta-Analyses) guidelines (Page et al., 2021) and was conducted in three stages: planning, execution, and summarization.

#### 2.1.1 Planning

A protocol was developed and discussed by the author team to guide the stages of the SR, incorporating essential information such as the article title, authors, objectives, keywords, research questions, sources of research, inclusion/exclusion criteria, search strings, selection of databases, and quality assessment of collected files. The questions to achieve the objective of the SR (**Table 1**) were formulated based on Population Intervention Comparison Results (PICOS) criteria (**Table 2**). The strategy employed was necessary to not only guide the questions but also to seek specific answers in order to exclude any biased responses.

**Table 1:** Questions guiding this SR.

Research questions	
Q1	What types of GPXs in plants?
Q1.1	Can GPXs have highly specific biological functions in plants? Which?
Q1.2	Where are GPXs located in plants?
Q2	What are the main methods used to determine the activity of GPXs?
Q3	What is the action mechanism of GPXs in plants?
Q3.1	Are GPXs involved in the interaction with other proteins in response to biotic stress?
Q3.2	Which proteins can plant GPXs interact with?
Q4	Does regulation of GPX expression protect plants from biotic stress?
Q5	What is the role of GPXs in plants as redox sensors?
Q6	Does regulation of GPX expression in transgenic plants help protecting against oxidative stress in defense against pathogens?
Q7	Can GPX control programmed death in plant cells as observed for animal GPXs?
Q8	Does selenium bioavailability increase the antioxidant potential of selenoprotein GPXs and non-selenoprotein GPXs?

**Table 2:** Criteria for delineation of the study, using the PICOS strategy.

	Description	Questions components
P	Population	Plant species under biotic stress
I	Intervention	Action of GPXs enzymes
C	Comparison	Plant species under biotic stress with and without the effect of the GPX action
O	Outcomes	GPXs favor the resistance or tolerance of plants against phytopathogens
S	Study	Scientific articles in English, peer-reviewed and experimental

#### 2.1.2 Execution

The searches for studies were conducted in the PubMed, Science Direct, and Springer pre-selected databases, which contain peer-reviewed journals aligned with the research theme. The string “glutathione peroxidase” AND “biotic stress” AND “cell death” AND “plant” was used for this search, with the Boolean connector AND used in the string to group keywords and main terms. The obtained files were imported in BIBITEX and MEDLINE formats to StArt and *R* program. After screening in *R* program, the files were transferred to StArt (v. Beta 3.0.3), where automated selection occurred based on the reading of titles, abstracts, and keywords. In this selection, inclusion and exclusion criteria were used (**Supplementary Table S1**).

#### 2.1.3 Summarization

The accepted studies, according to the inclusion criteria, were read in their entirety. This stage involved the extraction and systematization of data that addressed the questions proposed in the SR, including the creation of tables and figures to represent the findings. Metadata related to the production and dissemination of scientific knowledge (such as collaboration between countries, institutions, authors of the studies, and words from the titles) were analyzed from the selected articles using the *R* program with the Bibliometrix package. The program was also used to construct a heatmap of differential protein accumulation from Log fold change (LogFC) values ​​provided by articles that used proteomic analysis as a study strategy. Only one of these articles was not included in the heat map because it did not provide the Log fold change (LogFC) value of the differentially accumulated protein (Sharma et al., 2007). The heatmap was constructed using the Complexheatmap package. The subcellular localization of GPX proteins, when not indicated in the studies, was predicted using the DeepLoc 1.0 platform (https://services.healthtech.dtu.dk/services/DeepLoc-1.0/, accessed on August 21, 2023).

### 2.2 Protein-protein interaction network of selected GPXs

The interaction network analysis was conducted to examine the biological functions in which the GPX proteins from the selected studies should be involved. For this purpose, a search for homologs in a model organism (*Arabidopsis thaliana*) was performed using the STRING server version 11.0 (https://string-db.org/) (Jensen et al., 2009). The protein sequences used and their respective identities with homologs are presented in the **Supplementary Table S2**. The proteins were analyzed using the following parameters: meaning of network edges - confidence; active interaction sources - text mining, experiments, databases, co-expression, neighborhood, gene fusion, and co-occurrence; minimum required interaction score - high confidence (0.700) in 50 interactions, significance level of 0.7; maximum number of connectors to revel the 1^st^ and 2^nd^ layers and no more than 50 interactions. The data generated in the network were downloaded in TSV format and transferred to Cytoscape version 3.7.2 (https://cytoscape.org/) and BiNGO plugin (https://apps.cytoscape.org/apps/bingo) tools.

### 2.3 Phylogeny of selected GPXs

The alignment and construction of a phylogenetic tree were performed using amino acid residue sequences of GPXs obtained from the selected studies, to identify similarities and differences between these proteins, as well as evolutionary relationships. For studies that solely provided *GPX* gene information, BLASTx (https://blast.ncbi.nlm.nih.gov/Blast.cgi, accessed on August 22, 2023) was used to obtain the corresponding or homologous FASTA sequences of amino acid residues (**Supplementary Table S3**). Alignment was obtained using the Clustal Omega server with the multiple alignment tool (https://www.ebi.ac.uk/Tools/msa/clustalo/, accessed on August 22, 2023). The phylogenetic tree was constructed using the Neighbor-Joining method with 1000 bootstraps (Saitou and Nei, 1987). The method was applied in the MEGA 7 software (Kumar et al., 2016). The Poisson correction method (Zuckerkandl and Pauling, 1965) was employed to calculate evolutionary distances, expressed in units of the number of amino acid substitutions per site. The analysis involved 29 sequences, and all positions containing gaps and missing data were removed.

## 3 Results

### 3.1 Quality, distribution, and flow of knowledge in the field of GPXs in biotic stress

**Figure 1:**
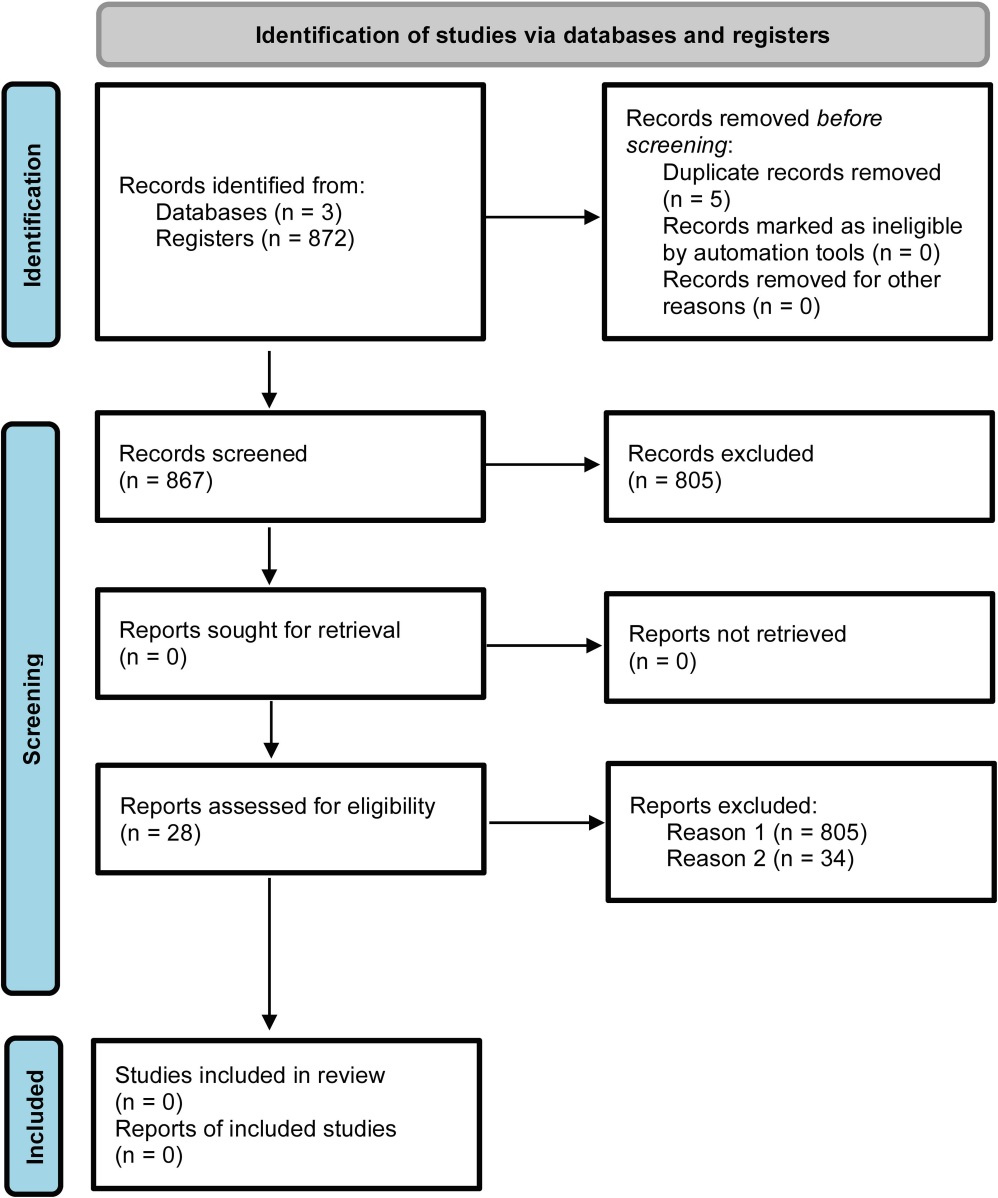
PRISMA flowchart depicting the identification and selection of studies on GPX responses to biotic stress and programmed cell death in plants.

**Figure 2:**
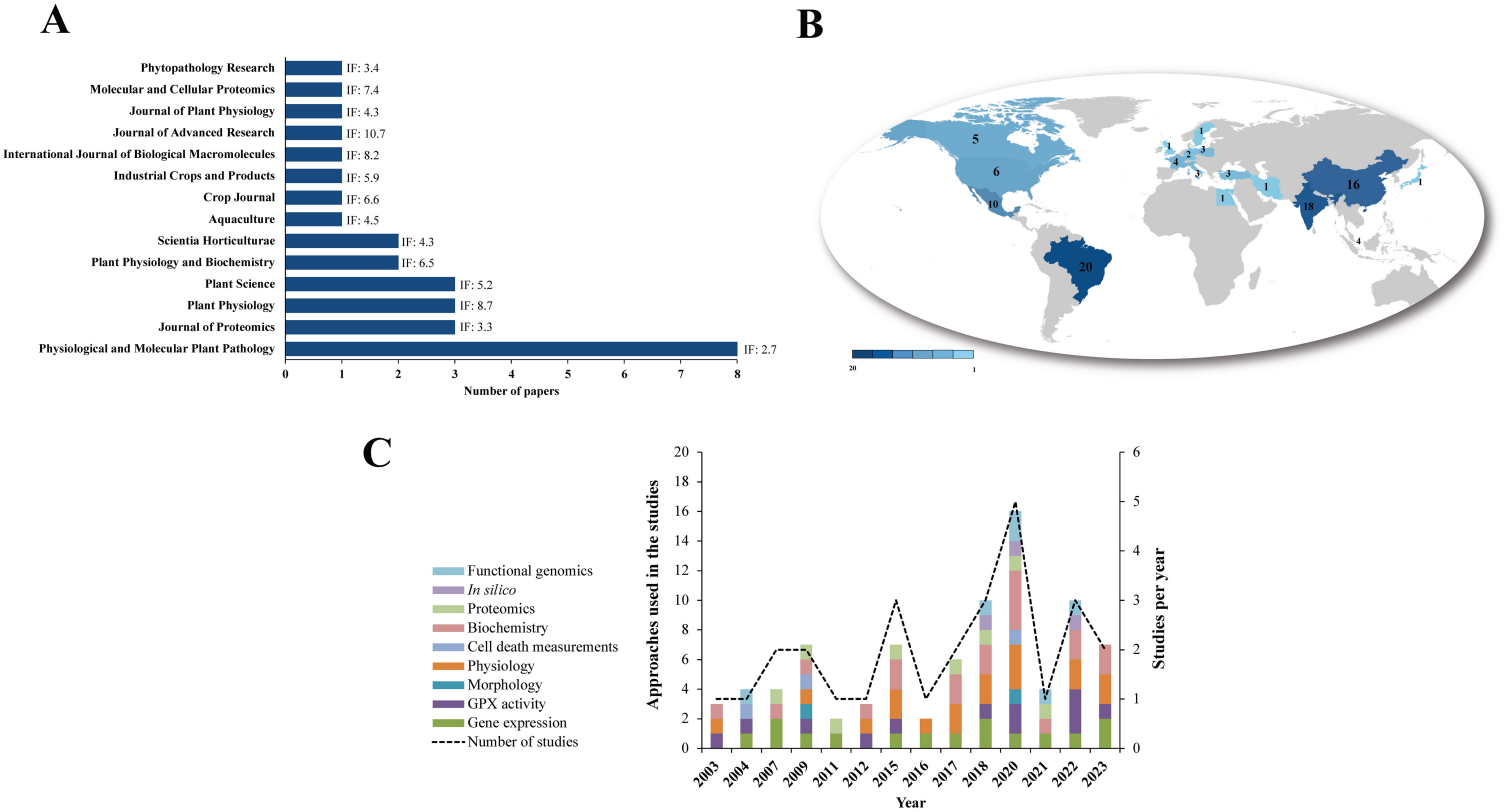
Bibliometric indicators of studies related to GPX response to biotic stress. **(A)** number of articles per journal, with their impact factor (IF). **(B)** Frequency of studies across countries. **(C)** Number of articles per year, with their respective study categories. * Searches for publications from the year 2023 were considered until March.

A total of 872 articles were retrieved from the Pubmed (32.8%), Science Direct (56.5%) and Springer (9.3%) databases. After excluding 5 duplicates in the StArt program, 805 articles were discarded based on title, abstract and keywords (**Figure 1**; **Supplementary Table S1**). The main reasons were: reviews (10%); book chapters (6%), animal GPXs (11%), GPXs-only approaches in abiotic stress (20%); duplicates (1%); and other unrelated topics (52%). Sixty-two articles were analyzed in full and 34 were excluded because they met the exclusion criteria, including studies on animal GPXs (6%), abiotic stress only (9%), duplicates (3%), reviews (3%) and other unrelated topics (79%). Finally, 28 articles were aligned with the study objectives and were considered eligible for this SR (**Figure 1**; **Supplementary Table S4**). The included studies were grouped according to the bibliometric data of the publishing journal and their respective impact factors (IF) (**Figure 2A**). They were published in journals with an IF between 2.7 and 10.7, with a focus on Physiological and Molecular Plant Pathology (8), Journal of Proteomics (3) and Plant Science (3), which had the highest number of articles among SR journal (**Figure 2A**). Most of the studies originated from Brazil (20%), India (18%) and China (16%) (**Figure 2B**) were published between 2003 and March 2023, with 2020 being the year with the highest number of studies (5) (**Figure 2C**). The 2020 articles addressed several techniques, such as gene expression, enzymatic activity, biochemistry and functional genomics. However, the number of articles did not show a constant increase, indicating that few studies have explored the potential of GPXs against biotic stresses, and consequently many questions remain unanswered about their role.

**Figure 3:**
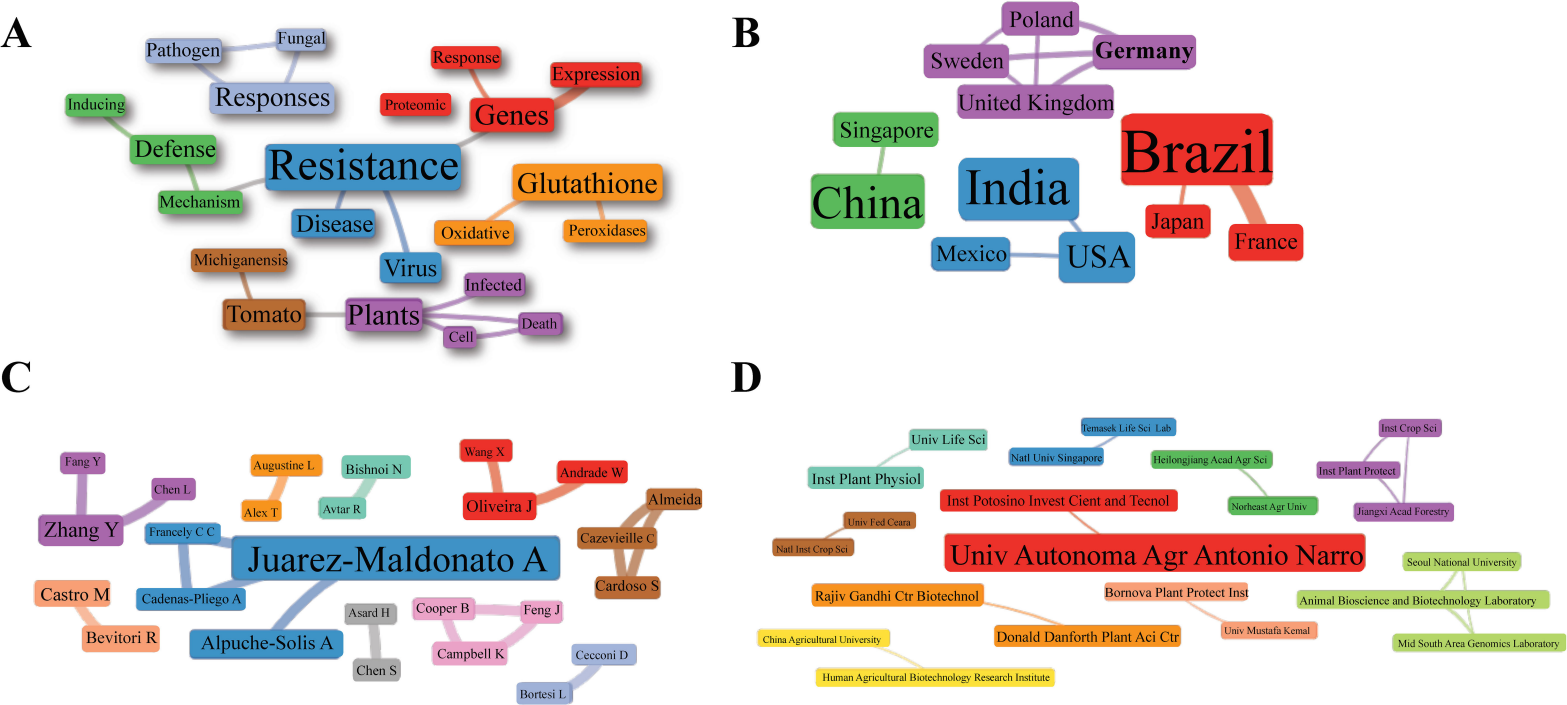
Networks for dissemination and collaboration of scientific knowledge on GPXs related to biotic stress. **(A)** Co-occurrence of words contained within journal titles. **(B)** Collaboration between countries of investigation. **(C)** Collaboration between authors. **(D)** Collaboration between institutions. Font sizes in the networks are proportional to the contribution of words, countries, authors, or institutions to the groups.

Collaboration and co-occurrence networks were constructed (**Figure 3**). Clusters were organized in different colors based on co-occurrence, with larger font size highlighting words that occurred more frequently within the studies selected in SR. The word network related to the research titles showed that “Peroxidases” and “Oxidative” appeared consistently associated with “Gluthatione” (in orange, **Figure 3A**). Collaborations were highlighted between Brazil, France and Japan (red cluster); India, USA and Mexico (blue cluster); China and Singapore (green cluster); and Germany, Poland, Sweden and the United Kingdom (purple cluster). Brazil, India and China represent the countries with the highest frequencies of research involving GPXs in biotic stress in plants (**Figure 3B**). The networks showing the collaborations between the main scientists of the selected articles and between the institutions were organized into ten clusters. The author “Juarez-Maldonato A” presented the highest number of collaborations (in blue, **Figure 3C**), while “Universidad Autonoma Agraria Antonio Narro” in Mexico was the most collaborative (in red, **Figure 3D**). Collaborations may favor more in-depth studies on GPXs in biotic stress, since they may involve different specializations and techniques. However, these collaborations are still limited to specific groups and expanding these partnerships beyond these limits can improve the quality of research and contribute to filling gaps on the role of GPXs in this context.

### 3.2 Plant GPXs participate in plant defense against pathogen-induced cell death

The types of GPXs addressed in the studies, and their association with different biotic stresses, are presented in **Table 3**. In 17.8% of the studies, plant GPXs are associated with cell death induced by different pathogens. The silencing of *GPX1* and *GPX7* genes in transgenic *A. thaliana* plants showed that these genes were involved in hypersensitive response (HR) during defense against *Pseudomonas syringae* bacteria (Chang et al., 2009), while the *GPX4*/*PHGPX* genes from *Nicotiana benthamiana* and *Lycopersicon esculentum*, were associated with defense against ferroptosis-type cell death and PCD induced by viral and fungal (necrotrophic) phytopathogens (Chen et al., 2004; Macharia et al., 2020). Transgenic *N. benthamiana* with silenced *GPX4* exposed to tobacco mosaic virus exhibited increased accelerated ferroptosis-type cell death compared to non-silenced control plants (Macharia et al., 2020). Moreover, transgenic tobacco leaves constitutively over-expressing *PHGPX* gene from *L. esculentum* (*LePHGPX*), inoculated with the necrotrophic fungi *Botrytis cinerea* and *Sclerotinia sclerotiorum*, were highly resistant compared to control leaves, which exhibited extended necrotic lesions (Chen et al., 2004). The transgenic tobacco leaves were also subjected to salt stress; the transient expression of *LePHGPX* in these stress-exposed leaves prevented DNA fragmentation and maintained membrane integrity. *LePHGPX* thus acted as cytoprotector in plants under different local stress conditions (Chen et al., 2004). Collectively, these data showed functional similarity to antiapoptotic *PHGPX* genes in animal organisms (Chen et al., 2004). Therefore, the *GPX4*/*PHGPX* genes in plants may be involved in defense against cell death induced by pathogens.

**Table 3:** Types of glutathione peroxidases (GPXs) proteins associated with biotic stress in plants, found in the selected studies.

Types of GPXs	Host plant species	Pathogen		Type of biotic stress	References
		Species	Type		
*cpAtGPX*s (*AtGPX1* and *AtGPX7*)	*Arabidopsis thaliana*	*Pseudomonas syringae*	Bacteria	Hypersensitive cell death	(Chang et al., 2009)
GPX unspecified	*Solanum melongena*	*Alternaria alternata*	Fungus	Spot disease	(Hassan et al., 2013)
HbGPX	*Hevea brasiliensis*	*Pseudocercospora ulei*	Fungus	South American Leaf Blight (SALB)	(Koop et al., 2016)
GPX (Glutp)	*Solanum tuberosum* White Lady	*Phytophthora infestans*	Oomycete	Late blight	(Hajianfar et al., 2016)
PHGPx	*Vigna unguiculata* (L.) Walp.	*Cowpea severe mosaic virus (CPSMV)*	Virus	Mosaic, chlorose, yellow patches, foliar distortion and leaf morphology alterations	(Varela et al., 2017)
TaGPX - TaGPX1 (A, A1, A2, B, D), TaGPX2-B, TaGPX3 (A, B, U), TaGPX4 (B, D, U), TaGPX5 (A, A2, B1, B2, D)	*Triticum aestivum*	*Puccinia striiformis* (Pst) *Blumeria graminis* (Bgt)	Fungus	Strip rust Powdery mildew	(Tyagi et al., 2018)
GPX unspecified	*Malus domestica*	*Colletotrichum gloeosporioides* (Penz.) Penz. & Sacc.	Fungus	Glomerella leaf spot (GLS)	(Rockenbach et al., 2015)
GPX unspecified	*Lycopersicon esculentum*	*Clavibacter michiganensis* subsp. *michiganensis* (Cmm)	Bacteria	Bacterial canker	(Soylu et al., 2003)
GPX15Hv	*Triticum aestivum* L.	*Barley Yellow Dwarf Virus* (BYDV)	Virus	Yellow dwarf virus (YDV) disease	(Wang et al., 2018)
NbGPX4	*Nicotiana benthamiana*	*Tobacco mosaic virus* (TMV)	Virus	Ferroptosis-like programmed cell death	(Macharia et al., 2020)
GPX unspecified	*Solanum lycopersicum* L. (cv. Santa Clara)	*Xanthomonas gardneri*	Bacteria	Bacterial spot disease	(Silveira et al., 2015)
GPX unspecified	*Solanum lycopersicum* (var. Pusa Ruby)	*Meloidogyne incognita*	Nematode	Root knot	(Khajuria and Ohri, 2020)
GSH-Px	*Brassica napus*	*Alternaria brassicae*	Fungus	Disease Alternaria black spot	(Sharma et al., 2007)
GPX unspecified	*Vitis vinifera*	*Plasmopara viticola*	Oomycete	Disease known as downy mildew	(Milli et al., 2012)
Phospholipid Glutathione Peroxidase	*Phaseolus vulgaris*	*Uromyces appendiculatus*	Fungus	Rust	(Lee et al., 2009)
GPX unspecified	*Ophiopogon japonicus*	*Yellow mosaic virus* (YMV)	Fungus	Yellow Mosaic Disease (YMD) or “Yellow plague of kharif pulses”	(Singh et al., 2020)
GPX4	*Solanum melongena* L.	*Verticillium dahliae*		*Verticillium* wilt	(Mahesh et al., 2017)
Probable phospholipid hydroperoxide glutathione peroxidase [*Oryza sativa* Japonica Group].	*Oryza sativa* L.	*Pyricularia oryzae (teleomorfo Magnaporthe oryzae*)	Fungus	Rice blast	(Távora et al., 2021)
GPX unspecified	Tomato “El Cid F1” *	*Clavibacter michiganensis*	Bacteria	Marginal necrosis in the leaves	(Cumplido-Nájera et al., 2019)
TcPHGPX, TcGPX2, TcGPX4, TcGPX6 and TcGPX8	*Theobroma cacao* L.	*Moniliophthora perniciosa*	Fungus	Witches’ broom disease	(Martins Alves et al., 2020)
LePHGPX	*Lycopersicon esculentum*	*Botrytis cinerea* and *Sclerotinia sclerotiorum*	Fungi	Programmed cell death	(Chen et al., 2004)
GPX unspecified	*Brassica napus* L.	*Sclerotinia sclerotiorum* (Lib.)	Fungus	Soft rot disease	(Yang et al., 2007)
GPX7	*Cucumis sativus* L.	*Alternaria cucumerina* (Ell et Ev.) Elliott.	Fungus	Alternaria leaf spot	(Sa et al., 2020)
GPX1, GPX2, GPX3	*Neoporphyra haitanensis*	*Vibrio mediterranei*	Bacteria	Yellow spot disease	(Zhu et al., 2023)
Five GPX (orthologs: phospholipid hydroperoxide glutathione peroxidase (*Zea mays*), glutathione peroxidase (GP) (*Nelumbo nucifera*), nuclear gene encoding chloroplast protein *(Zantedeschia aethiopica*), Glutathione peroxidase (*Zea mays*) and Glutathione peroxidase (*Solanum lycopersicum*)	*Zingiber zerumbet*	*Pythium myriotylum*	Oomycete	Soft rot disease	(Alex et al., 2022)
GPX unspecified	*Solanum lycopersicum* L.	*Fusarium oxysporum*	Fungus	Vascular wilt disease and crown and root rot	(González-García et al., 2022)
GPX unspecified	*Brassica juncea* (L.) Czern & Coss.	*Sclerotinia sclerotiorum (Lib.)*	Fungus	Sclerotinia stem rot	(Singh et al., 2022)
GPX unspecified	*Salviae miltiorrhizae*	*Aphis gossypii*	Aphids	Chlorosis and cell death	(Zhang et al., 2023)

* “El Cid F1”: indeterminate growth tomato variety (Harris Moran, Davis, CA, USA) of the saladette type.

### 3.3 Plant resistance conferred by upregulation of *GPX* gene expression and/or GPX protein accumulation depends on pathogen type encountered during the interaction

**Figure 4:**
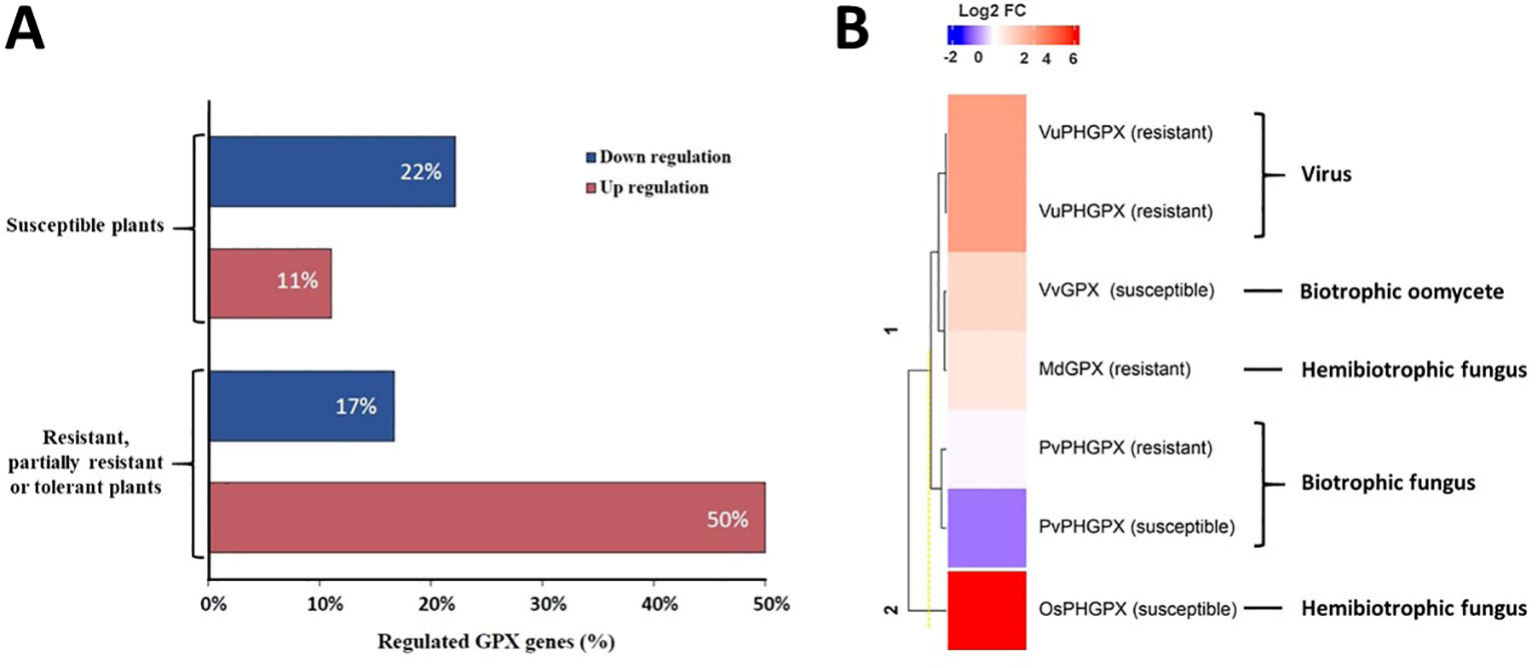
Studies on of plant GPX regulation in response to pathogens. **(A)** Percentage of studies with *GPX* genes differentially regulated in resistant or partially resistant or tolerant plants compared to susceptible ones, in the presence of pathogens. **(B)** Heatmap of the differential accumulation of GPX proteins in plants in interaction with pathogens. The differential accumulation of proteins is represented by the Log fold change (LogFC) value provided by the selected studies; only one of the studies is not included in the heatmap, as this value was not provided in the available information. The following GPXs are represented: VuPHGPX (PHGPX from *Vigna unguiculata*) in an incompatible interaction with Cowpea severe mosaic virus (CPSMV); MdGPX (GPX from *Malus domestica* cv. Fuji) in an incompatible interaction with *Colletotrichum gloeosporioides*; VvGPX (GPX from *Vitis vinifera*) in a compatible interaction with *Plasmopara viticola*; PvPHGPX (PHGPX from *Phaseolus vulgaris*) in a compatible interaction with *Uromyces appendiculatus*; OsPHGPX (PHGPX from *Oryza sativa* L.) in a compatible interaction with *Magnaporthe oryzae*.

The evaluation of GPX expression in response to biotic stress was used in 42.9% of the studies (**Table 3**). *GPX* genes were upregulated in 50% of the studies in resistant, partially resistant or tolerant plants, and in 11% of the studies in susceptible plants. Downregulation was observed in 22% of the studies with susceptible plants and in 17% of the studies with resistant, partially resistant or tolerant plants (Chen et al., 2004; Yang et al., 2006; Chang et al., 2009; Hajianfar et al., 2016; Koop et al., 2016; Tyagi et al., 2018; Wang et al., 2018; Macharia et al., 2020; Martins Alves et al., 2020; Sa et al., 2020; Zhu et al., 2023). These data indicate that, although resistant or tolerant plants present a higher frequency of upregulation of *GPX* genes, this induction is not a trend exclusive to them, being also observed in susceptible plants (**Figure 4A**).

GPX accumulation in plants varies according to several factors, including the pathogen’s lifestyle, as demonstrated by a study of this RS (Sharma et al., 2007; Lee et al., 2009; Milli et al., 2012; Rockenbach et al., 2015; Varela et al., 2017; Távora et al., 2021). Pathogen infection induces oxidative stress in cells, increasing ROS levels, which can activate antioxidant pathways, such as increased GPX activity. Depending on the pathogen’s lifestyle, modulation of ROS levels can promote the death of infected tissues, restricting infection or favoring susceptibility. RS studies that used proteomic analysis (25%, **Table 3**) confirmed the differential accumulation of GPXs in several plant species during infection (Sharma et al., 2007; Lee et al., 2009; Milli et al., 2012; Rockenbach et al., 2015; Varela et al., 2017; Távora et al., 2021). Among these studies, a comparative proteomic analysis in susceptible grapevine leaves (*Vitis vinifera*) inoculated with the biotrophic oomycete *Plasmopora viticola* revealed upregulation in all proteins associated with oxidative stress. Among them, GPX (VvGPX) showed a notable increase in expression, particularly 94 hours post inoculation (hpi) (Milli et al., 2012). Another study on compatible interaction between the plant *Oryza sativa* L. and the hemibiotrophic pathogen *Magnaporthe oryzae* identified a significant increase in proteins related to oxidative stress. Among them, PHGPX (OsPHGPX) showed marked upregulation in the first 12 hpi (Távora et al., 2021). In a study in common bean leaves (*Phaseolus vulgaris*) resistant to race 49 and susceptible to race 41 of *Uromyces appendiculatus*, protein levels were evaluated at 24 and 72 hpi with these biotrophic fungi. At 24 hpi, results showed reduced accumulation of PHGPX (PvPHGPX) in leaves susceptible to race 41. However, at 72 hpi, PHGPX accumulation levels were comparable between leaves inoculated with both races (41 and 49) (Lee et al., 2009). One study investigated *Colletotrichum gloeosporioides* infection in apple (*Malus domestica*) leaves of resistant and susceptible cultivars. Proteomic analyses performed at 48 hpi revealed accumulation of MdGPX in the resistant cultivar, indicating an adaptive response to the hemibiotrophic fungus. Another study investigated the proteomic responses in two *Brassica napus* lines (tolerant and susceptible) challenged by the necrotrophic pathogen *Alternaria brassicae*. At 48 hpi, the accumulation of GPX (BnGPX) and other proteins related to free radical detoxification was observed, especially in the tolerant line, highlighting its importance to biotic stress. Similarly, a study on cowpea (*Vigna unguiculata*) in an incompatible interaction with cowpea severe mosaic virus (CPSMV) reported a significant increase in the abundance of two types of chloroplastic PHGPX (VuPHGPX) six days post inoculation (dpi). These results suggest that susceptibility and resistance, related to the greater or lesser accumulation of GPX, are directly influenced by the type of pathogen interacting with the host plant (**Figure 4B**).

### 3.4 Various biological functions are linked to plant GPXs

**Figure 5:**
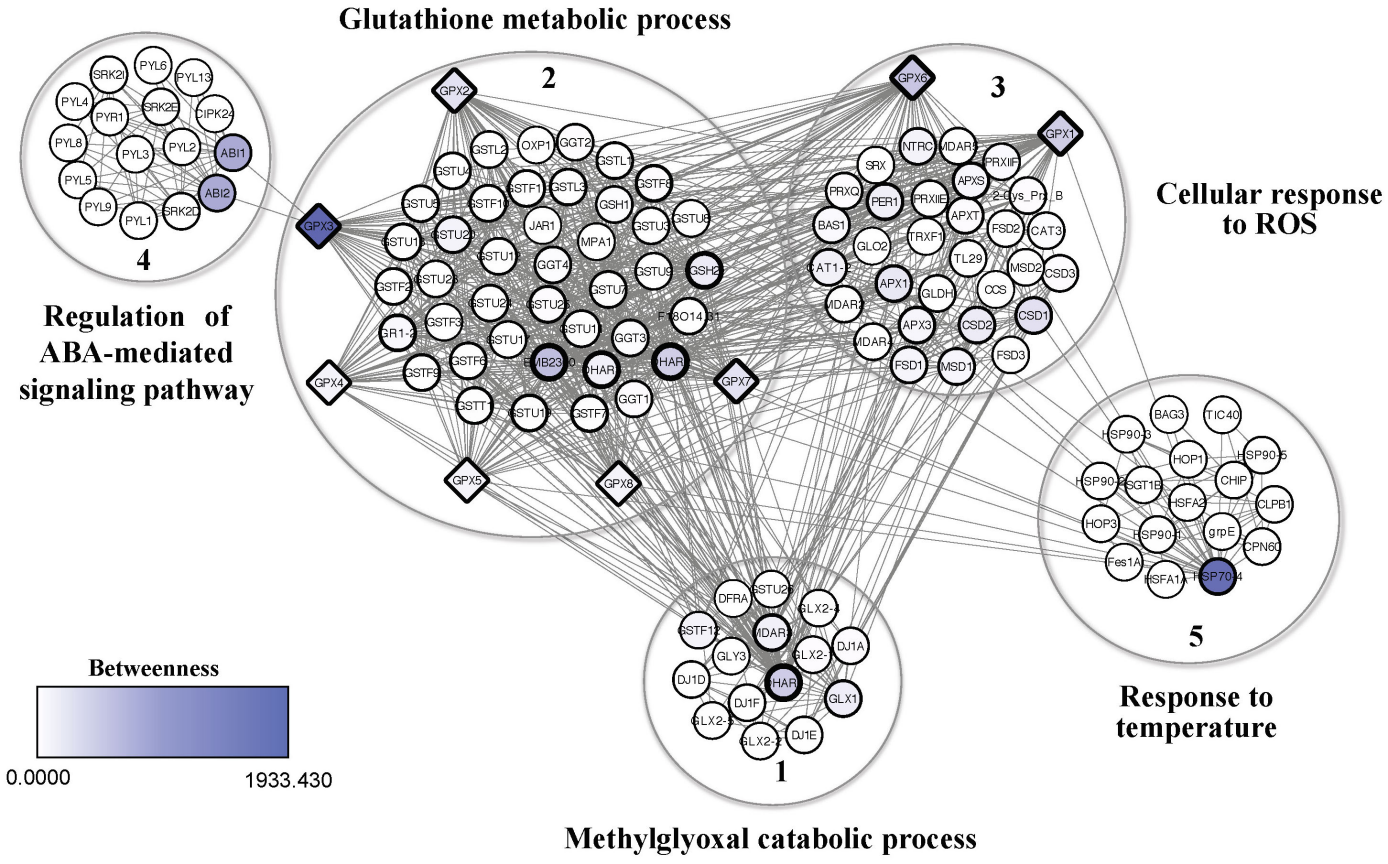
Interaction network of GPX proteins extracted from SR studies, using homologous proteins from the model organism *Arabidopsis thaliana*. Protein-protein interaction network was generated using the String plugin, Cytoscape, and BiNGO. Colors ranging from white to shades of blue represent betweenness values, with more intense colors indicating higher values and representativeness among clusters. Representative biological processes in the network include: methylglyoxal catabolic process (cluster 1), glutathione metabolic process (cluster 2), cellular response to ROS (cluster 3), regulation of the ABA-mediated signaling pathway (cluster 4), response to temperature (cluster 5).

The protein-protein interaction network of plant GPXs obtained from the analyzed studies, was generated from *A. thaliana* orthologous proteins (**Figure 5**; **Supplementary Table S2**). The network resulted in 127 proteins (nodes), with 1017 connectors, forming 5 clusters, with a high level of confidence (0.7). BiNGO analysis revealed the most representative biological processes within the network: catabolic process of methylglyoxal (cluster 1); glutathione metabolic process (cluster 2); cellular response to ROS (cluster 3); regulation of the abscisic acid (ABA)-mediated signaling pathway (cluster 4); and response to temperature (cluster 5) (**Figure 5**).

The highest betweenness values – indicating high connectivity between clusters – were represented by darker shades of blue, while the highest degree values (node degree) – reflecting a high number of connections – were represented by thicker node (proteins) borders (**Figure 5**). Thus, the GPX proteins in the network, especially GPX3, showed high betweenness values, acting as bottlenecks with a high capacity of interaction and/or signaling within the network functions. GPX proteins also showed high degree values, indicating a large number of connections, and characterizing them as hubs with important regulatory functions within the network (**Figure 5**). The GPX2, GPX3, GPX4, GPX5, GPX7 and GPX8 found in the cluster 2 were directly associated with glutathione metabolic processes, while GPX1 and GPX6, along with other proteins, formed the cluster 3 and were associated with cellular responses to ROS. However, all GPXs were directly and indirectly associated with other processes in the network (**Figure 5**). GPX3 directly interacted with the ABI1 and ABI2 phosphatases in cluster 4. The GPX1, GPX2, GPX3, GPX4, GPX5, GPX6, GPX7, and GPX8 were linked with a glutathione reductase proteins (EMB2360) in cluster 2, with glutathione transferases (DHAR1 and 2) in clusters 1 and 2, with a heat shock protein (HSP70-4) in cluster 5, and with a superoxide dismutase (CSD1) in cluster 3 (**Figure 5**). These interactions were related to specific processes such as regulation of glutathione level, ROS elimination, protein folding and translocation into organelles, and degradation of damaged proteins under stress.

### 3.5 The induction of GPX activity in plants provides protection against biotic stress

**Figure 6:**
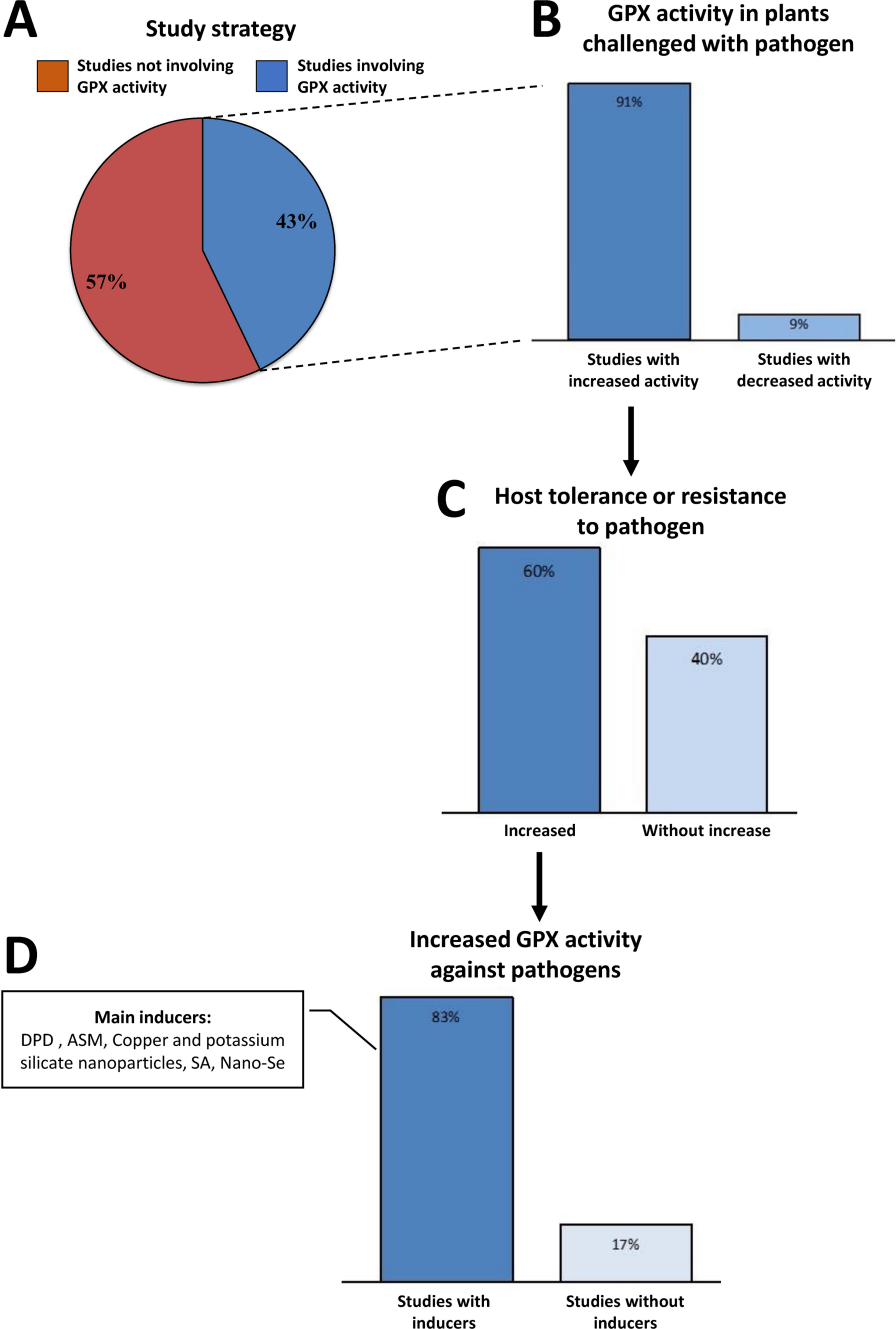
Studies that employed GPX activity analysis as a strategy for defense against pathogens. **(A)** Percent of studies involving or not GPX activity. **(B)** Studies containing GPX activity in plants challenged with pathogen. **(C)** Studies related to GPXs involved in host tolerance or resistance to pathogen. **(D)** Studies showing increased GPX activity against pathogens with or without inducers. ASM: acibenzolar-S-methyl (benzo (Pereira, 1989; Blomme et al., 2017; Moreira et al., 2022) thiadiazole-7-carbothioic acid S-methyl ester); DPD, 2-amino 4-6-dimethyl pyridine; SA, salicylic acid; Se, selenium.

The determination of antioxidant enzyme activity is often used to evaluate responses of susceptible and/or resistant plants against pathogens. Among the analyzed studies, 43% employed the strategy of analyzing GPX activity in response to biotic stress (**Figure 6A**). Among them, 91% of the studies showed increased GPX activity in plant challenged with bacteria, fungi, viruses, nematodes, or aphids (**Figure 6B**; **Supplementary Table S5**). Induction of this activity promoted increased tolerance or resistance in 60% of these studies (**Figure 6C**).

In studies demonstrating increased tolerance or resistance, 83% employed exogenous molecules to stimulate plant resistance, thereby resulting in elevated GPX activity. These molecules acted as inducers of enzyme efficiency in detoxification and cell protection (**Figure 6D**). The infection of *Solanum melongena* (eggplant) by the fungus *Alternaria alternata* led to an inhibition of plant antioxidant protein activities, including GPX one, promoting the plant susceptibility to the disease. However, when eggplants were treated with a pyrimidine derivative (DPDP) and then challenged with *A. alternata*, the inhibition of antioxidant activities was reversed. Thus, DPDP increased the activities of antioxidant enzymes, including GPX, enhancing the efficiency of ROS elimination by the host, and consequently inhibiting disease progression (Hassan et al., 2013) (**Supplementary Table S3**). In another study, the inducer acenbenzolar - 5 methyl (ASM) was tested against bacterial canker in tomato: the use of ASM significantly and gradually boosted the activities of peroxidases (POX) and GPX, particularly evident when the treated plants were subjected to *Clavibacter michiganensis* subsp. *michiganensis* (Cmm). ASM treatment amplified the GPX activity, thereby fortifying defense against oxidative damage linked to biotic stress induced by Cmm (**Supplementary Table S3**) (Soylu et al., 2003). The tolerance of tomato plants to Cmm was also assessed under treatment with copper and potassium silicate nanoparticles (Cumplido-Nájera et al., 2019). Treatment with both nanoparticles resulted in a 28% reduction in Cmm disease severity, and when considering enzymatic activity, application of copper nanoparticles (at high concentration) together with low or high doses of silicate, resulted in a notable augmentation of GPX activity by 161% and 191%, respectively, compared to controls. Ascorbate peroxidase (APX) activity was also induced. These associations suggest that nanoparticles collectively stimulate antioxidant activity, thereby enhancing the tomato plants’ ability to tolerate oxidative stress induced by Cmm, resulting in reduced pathogen severity and concurrently, lower yield loss (Cumplido-Nájera et al., 2019) (**Supplementary Table S3**). The effects of exogenous addition of salicylic acid (SA) were evaluated on the resistance of *Neoporphyra haitanensis* to *Vibrio mediterranei* infection, which causes symptoms of yellow spot (Zhu et al., 2023). Antioxidant enzyme activities, such as GPX, increased sharply in proportion to the SA concentration compared to the untreated control. Although it did not inhibit the growth of pathogenic bacteria, SA-treated plants were significantly healthier than the controls. SA may have triggered the antioxidant defense system by inducing high levels of H_2_O_2_, signaling the plant to enhance redox regulation efficiency (Zhu et al., 2023) (**Supplementary Table S3**). Among the studies analyzed, only one associated selenium (Se) interference with GPX enzyme activity in plants subjected to biotic stress. In this study, the effects of nano-Se on *Salviae miltiorrhizae* growth and defense responses was investigated (Zhang et al., 2023): antioxidant enzyme activities, including GPX, were induced with treatments of 10 mg/l and 20 mg/l of nano-Se. Furthermore, aphid infestation caused chlorosis and cell death in untreated *S. miltiorrhizae* leaves, whereas these symptoms were absent in plants treated with nano-Se (Zhang et al., 2023) (**Supplementary Table S3**).

Studies employed different strategies to detect GPX activity (**Supplementary Table S3**). In 54% of cases, peroxide reduction by GPX was assessed via the glutathione or thioredoxin pathways, based on NADPH oxidation (Soylu et al., 2003; Chang et al., 2009; Hassan et al., 2013; Silveira et al., 2015; Singh et al., 2020; Singh et al., 2022). GPX activity was also measured using the absorbance method for oxidized glutathione (420 nm) (Khajuria and Ohri, 2020), or by the absorbance method at 412 nm using the DTNB reagent (5,5′-dithiobis-(2-nitrobenzoic acid) that binds reduced glutathione; therefore, the decrease in absorbance may reflect GPX activity (**Supplementary Table S3**) (Cumplido-Nájera et al., 2019; González-García et al., 2022).

### 3.6 Plant GPX proteins associated with biotic stress exhibit evolutionary relationships

**Figure 7:**
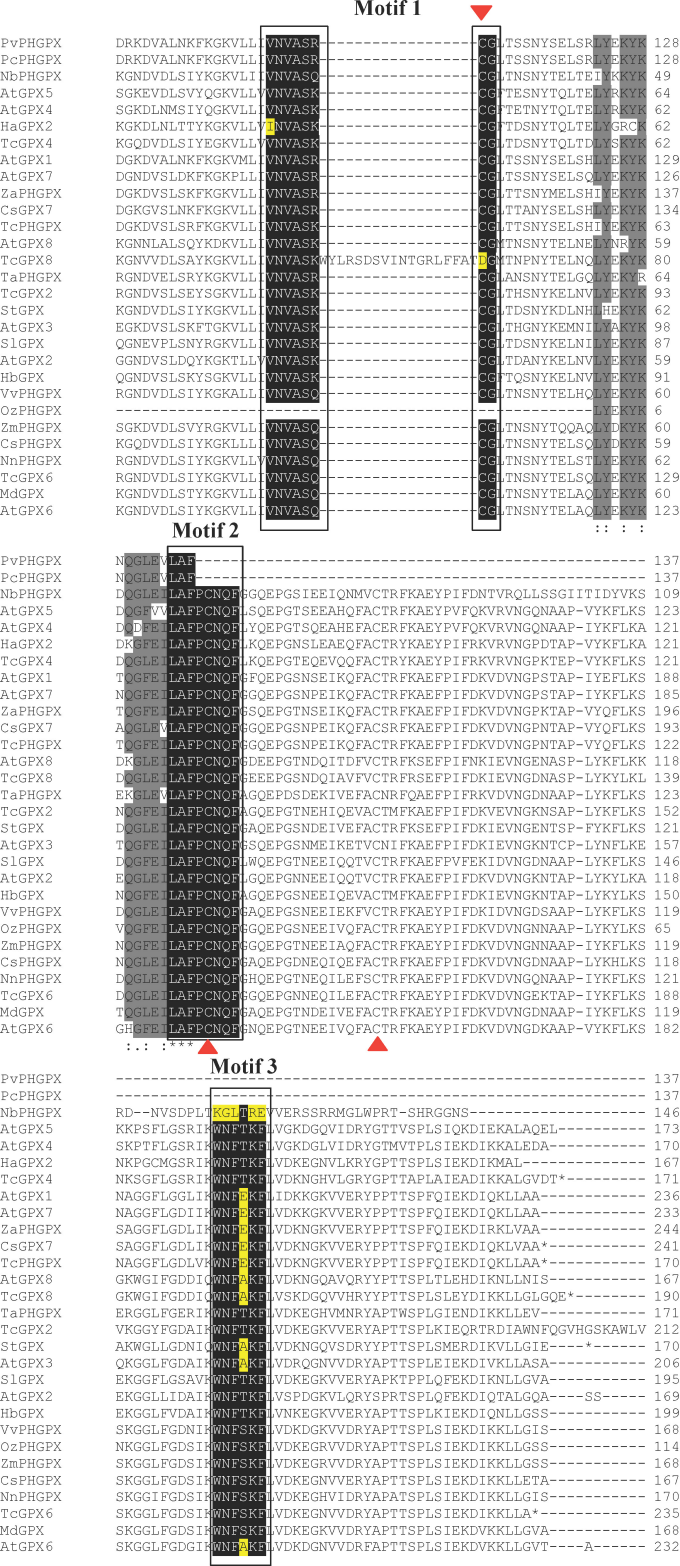
Alignment of the amino acid sequence of GPXs obtained from the selected studies of this SR. Black highlights indicate motifs 1, 2, and 3, which are common in plant GPXs. Yellow highlights indicate amino acids that differed within the conserved motifs, and gray highlights indicated the main conserved regions outside the motifs. Red triangles indicate conserved cysteine residues.

The alignment of GPX amino acid residue sequences was performed using sequences from eligible studies of this SR (**Figure 7**; **Supplementary Table S5**). The comparison revealed three frequent conserved motifs in plant GPXs (**Figure 7**). Within these motifs, some sequences showed alterations, such as: in GPX2 homolog of *V. unguiculata* (HaGPX2), a substitution of an amino acid residue (isoleucine for valine) was identified in motif 1, in GPX8 of *Theobroma cacao* (TcGPX8), a substitution of an amino acid residue (cysteine for aspartate) occurred in the same motif, and in PHGPX of *N. benthamiana* (NbPHGPX), a nearly complete alteration in motif 3 was revealed, conserving only one amino acid residue (**Figure 7**). Conserved amino acid residues among GPXs, outside of the motifs, were detected and are highlighted in gray. Conserved cysteine amino acid residues, characteristic of plant proteins, were demonstrated among the sequences and are marked with red triangles (**Figure 7**).

**Figure 8:**
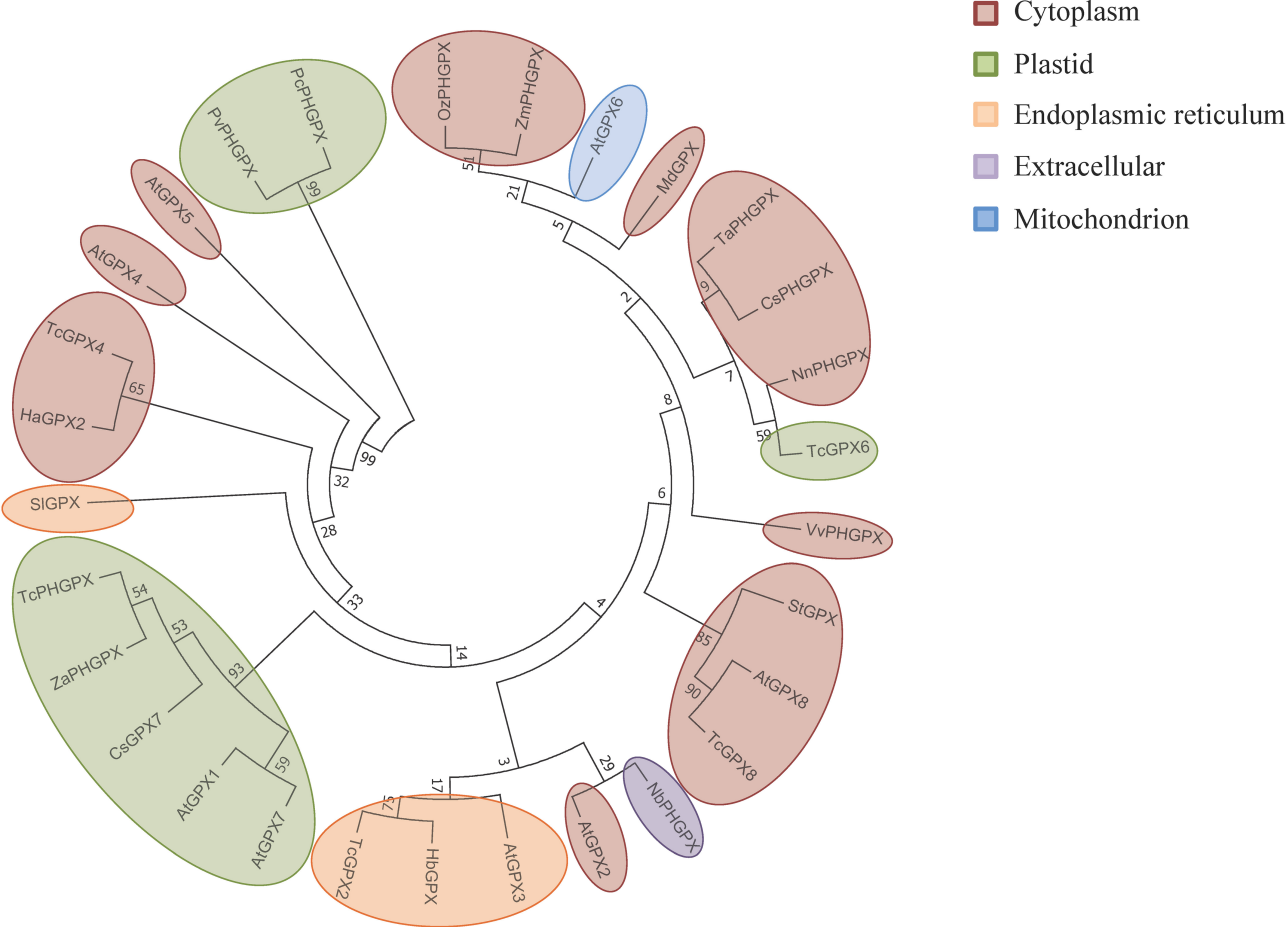
Evolutionary relationship between the GPXs obtained from the selected studies of this SR. The phylogenetic tree was generated using the neighbor-joining method with alignment in Clustal W with 1000 bootstraps, conducted in MEGA7. GPXs are highlighted in different colors according to the subcellular localization predicted by DeepLoc-1.0.

The phylogenetic analysis of sequences revealed evolutionary relationships among GPX proteins from *T. cacao* (PHGPX), *Zingiber zerumbet*, *Cucumis sativus* L., and *A. thaliana* (GPX1 and GPX7) (Chang et al., 2009; Martins Alves et al., 2020; Sa et al., 2020; Alex et al., 2022). These proteins formed the same clade and exhibited chloroplastic subcellular localization (**Figure 8**; **Supplementary Figure S1**). The GPX4 of *T. cacao* and the homologous GPX2 of *V. unguiculata* also clustered together and displayed the same cytoplasmic localization pattern (Varela et al., 2017; Martins Alves et al., 2020). In another clade, GPX proteins from *Solanum tuberosum*, GPX8 from *A. thaliana*, and *T. cacao* also showed evolutionary relationships, sharing the same chloroplastic localization (Chang et al., 2009; Hajianfar et al., 2016; Martins Alves et al., 2020). The GPX3 of *A. thaliana*, GPX of *Hevea brasiliensis*, and GPX2 of *T. cacao* formed a phylogenetic group with subcellular localization in the endoplasmic reticulum (Chang et al., 2009; Koop et al., 2016; Martins Alves et al., 2020).

## 4 Discussion

### 4.1 Plant GPXs protect cells against cell death, just like animal GPX4s/PHGPX

Pathogen infection induces oxidative stress in plants, leading to ROS accumulation that disrupts membrane integrity through lipid peroxidation (Keppler et al., 1989; Torres et al., 2006), destabilizing cellular homeostasis and amplifying stress responses (Sharma et al., 2012; Farmer and Mueller, 2013; Kuźniak and Gajewska, 2024). Lipid peroxidation products can activate cell death pathways, influencing pathogen progression (Dey et al., 2020). Antioxidant enzymes like GPXs are crucial in mitigating these damaging effects. GPXs decompose peroxides, preventing their interaction with membrane lipids and averting successive damage (Rouhier and Jacquot, 2005; Dietz, 2011). Among these, phospholipid hydroperoxide glutathione peroxidases (PHGPX), also known as GPX4 in mammals, reduce lipid hydroperoxides to alcohols using glutathione as an electron donor, protecting cell membranes from oxidative damage and cell death (Ursini et al., 1995; Takebe et al., 2002; Imai and Nakagawa, 2003; Nakagawa, 2004). This isoform is conserved in plants, where it exhibits functional similarity to animal GPX4. For instance, LePHGPX in transgenic tobacco plants protected against DNA fragmentation, membrane integrity loss, and oxidative stress induced by necrotrophic fungi, mirroring anti-apoptotic roles observed in animals (Chen et al., 2004).

Ferroptosis, a form of iron-dependent lipid peroxidation-mediated cell death, was first described in animals and is regulated by GPX4 (Dixon et al., 2012; Seibt et al., 2019; Feng et al., 2020; Chen et al., 2021; Ma et al., 2022). In plants, ferroptosis also occurs during pathogen infections (Distéfano et al., 2017), as demonstrated in *N. benthamiana* infected with the tobacco mosaic virus (TMV 24A/UPD) (Macharia et al., 2020). Silencing GPX4 accelerated this cell death, highlighting its protective role. Interestingly, ferroptosis may function as a plant defense mechanism, enhancing resistance to pathogens such as oomycetes. This parallels the emerging therapeutic potential in animals, where GPX4-regulated ferroptosis is being explored as a strategy for treating diseases such as cancer (Friedmann Angeli et al., 2014; Yang et al., 2014; Viswanathan et al., 2017).

Plant GPXs share homology with mammalian GPX4 (Criqui et al., 1992; Holland et al., 1993; Sugimoto and Sakamoto, 1997; Yang et al., 2005; Navrot et al., 2006), but they generally lack the selenocysteine (SeCys) residue critical for the high catalytic efficiency of animal GPXs, substituting it with cysteine (**Figure 7**) (Herbette et al., 2007; Bela et al., 2015). Despite this structural difference, plant GPXs exhibit catalytic efficiencies comparable to animal GPXs (i.e. ranging between 10^-3^ to 10^6^ M^-1^s^-1^ in plants and between 10^-7^ to 10^8^ M^-1^s^-1^ in animals) (Dietz, 2011). Recent studies have highlighted their functional relevance. For example, PHGPX from *O. sativa* efficiently reduces phospholipid hydroperoxides using glutathione or thioredoxin (Wang et al., 2007), while the *T. cacao* PHGPX protects against H_2_O_2_ accumulation, membrane damage, and cell death induced by fungal proteins in *N. benthamiana* cell suspensions (do Carmo Santos et al., 2024). Although plant GPXs lack the catalytic advantage conferred by SeCys, their protective roles against oxidative stress and regulation of cell death underscore their importance in plant defense (Maiorino et al., 1995; Mannes et al., 2011). The functional similarity to animal GPX4 suggests a conserved evolutionary mechanism linking oxidative stress mitigation and cell death regulation in both kingdoms.

### 4.2 Regulation, function, and evolutionary insights of plant GPX isoforms in relation to stresses

Gene expression analysis has demonstrated that GPX isoforms play distinct roles in plant stress responses, exhibiting differential regulation based on the type of stress encountered (**Figure 4A**). For instance, in *Panax ginseng*, GPX1 expression increased 8.6-fold at 48 hpi with the hemibiotrophic fungus *C. gloeosporioides*. Conversely, GPX2 expression decreased during the same interaction (Kim et al., 2014). This suggests that GPX1 might be critical in mitigating oxidative stress during the early stages of pathogen challenge, while GPX2 might have a reduced role in biotic stress defense. In *Camellia sinensis*, GPX2 was unresponsive to herbivore attacks but was strongly induced (3.3- to 4.9-fold) following treatments with the phytohormones gibberellic acid (GA) and methyl jasmonate (MeJA). This indicates that CsGPX2 responds primarily to abiotic stresses and developmental signals rather than biotic challenges (Fu, 2014). Additional evidence supports the association of certain GPX isoforms, such as GPX2, with abiotic stress and developmental processes. For instance, HvGPX2 in *Hordeum vulgare* is induced under salinity, drought, and oxidative herbicide stress, while AtGPX2 in *A. thaliana* is expressed during key developmental stages, including root elongation, leaf expansion, and flowering (Churin et al., 1999; Chen et al., 2011; Islam et al., 2015; Zhou et al., 2018).

The alignment of GPX amino acid residues under biotic stress revealed conserved cysteine residues essential for catalytic activity within motifs 1 and 2 (red triangles, **Figure 7**). These residues enable the reduction of peroxides, with key cysteines forming intramolecular disulfide bridges to maintain redox balance. Variations in conserved motifs, such as in *T. cacao* GPX8 (*TcGPX8*), *V. unguiculata* GPX2 (*VuGPX2*), and *N. benthamiana* PHGPX (*NbPHGPX*), suggest potential functional differences. These differences warrant experimental studies to determine how they impact GPX activity in biotic stress (Boschi-Muller et al., 2000; Herbette et al., 2002).

**Figure 9:**
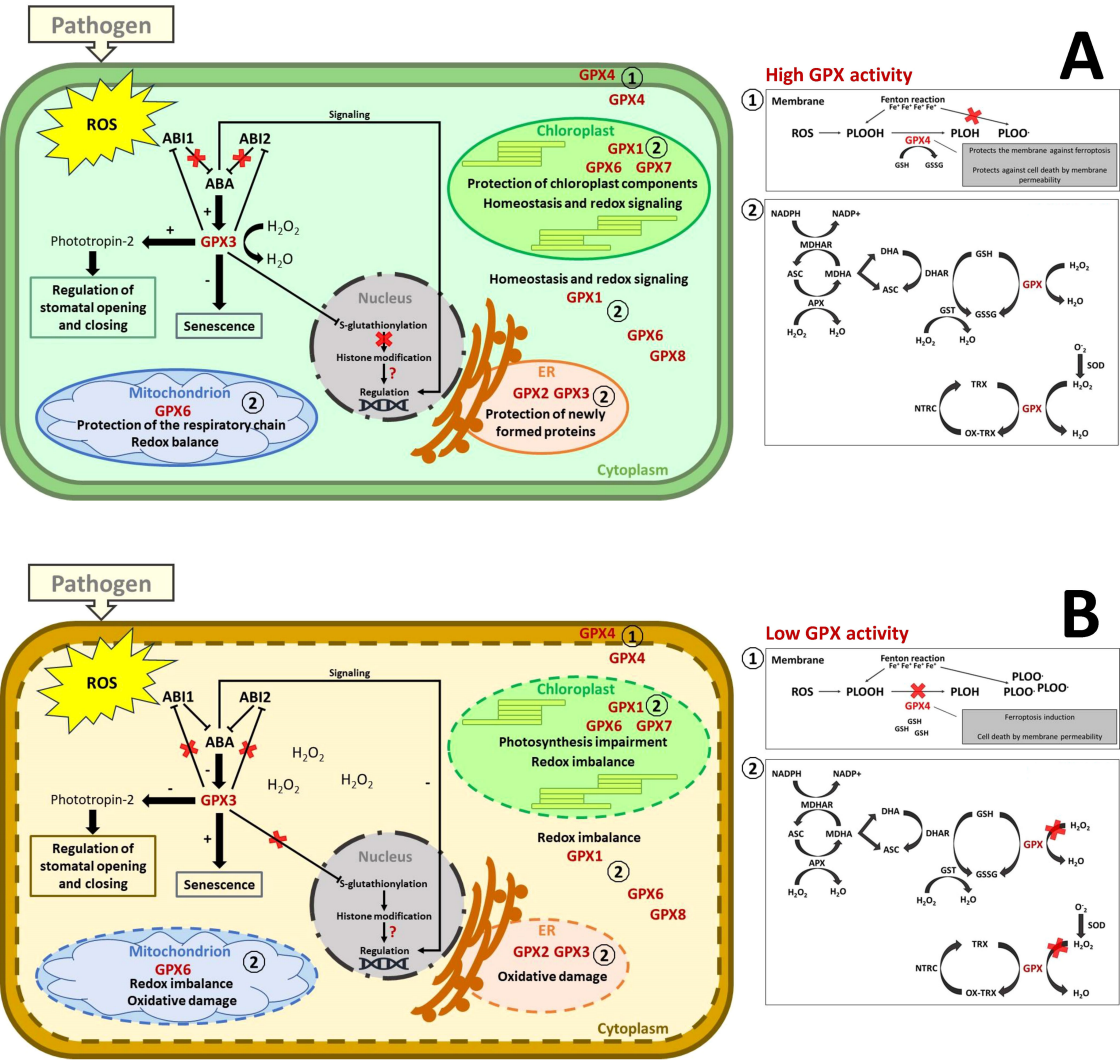
Plant GPX responses under pathogen-induced stress. **(A)** Representation of the processes associated with high GPX accumulation or activity. GPX1, GPX2, GPX3, GPX5, GPX6, GPX7, and GPX8 are part of the antioxidant complex, maintaining a balance between the production and reduction of ROS. Heightened stress, such as that triggered by pathogens, can boost GPX activity, maintaining ROS levels adequate for defense signaling without causing damage to organelle function or compromising their structural integrity. Some components of the enzymatic and non-enzymatic antioxidant system are represented: superoxide dismutase (SOD); glutathione peroxidase (GPX); ascorbate peroxidase (APX); dehydroascorbate reductase (DHAR); monodehydroascorbate reductase (MDHAR); NADPH-dependent thioredoxin reductase (NTRC); ascorbate (AsA); monodehydroascorbate (MDHA); dehydroascorbate (DHA); glutathione (GSH); glutathione disulfide (GSSG); reduced (TRX) and oxidized (OX-TRX) forms of thioredoxin. GPX can use both thioredoxin and reduced glutathione as electron donors for antioxidant activity. Cytoplasmic GPX4 is involved in defense by protecting membranes from cell death through ferroptosis or other forms of cell death due to membrane permeability independent of iron. GPX3, in addition to its antioxidant processes, may inhibit phosphatase 2C (PP2C), ABI1 and ABI2, which are inhibitors of ABA signaling. Once inhibited by GPX3, ABA signaling increases, potentially regulating other processes such as GPX3 accumulation itself, which reduces senescence through detoxification, serving as a defense mechanism to keep cells alive, and gene regulation. Additionally, GPX3 can induce the accumulation of phototropin-2, which is key to regulating stomatal opening and closure, potentially acting as a physical barrier to pathogen invasion. Another process that this enzyme may be involved in is the suppression of accumulation and post-translational modification through S-glutathionylation, which controls histone modification and may affect epigenetic regulation. However, maintaining redox balance within the cell can be crucial for preventing infection progression, except for pathogenic organisms that require living cells to develop, such as biotrophs. **(B)** Representation of the processes associated with low GPX accumulation or activity. Reduced GPX activity or accumulation in response to pathogens may be associated with the progression of lipid peroxidation, leading to destabilization of membrane structure followed by cellular permeability. Furthermore, it can lead to increased senescence due to reduced antioxidant activity. The imbalance between ROS production and GPX detoxification can compromise organelle components, leading to malfunction, such as reduced photosynthesis and respiratory chain activity. Cellular collapse can increase susceptibility to necrotrophic pathogens but deter biotrophic pathogens. The isoforms shown in this figure are GPXs from *Arabidopsis thaliana*, homologous to those reported in the SR articles (see **Supplementary Table S2**).

However, global phylogenetic analysis reveals GPXs have diverged across species while retaining their functional core. *T. cacao* GPXs clustered separately from *A. thaliana* GPXs, indicating evolutionary divergence despite all GPXs descending from a common ancestor (**Figure 8**). However, *TcGPX* shares clades with plant species like *C. sinensis* and *E. grandis*, reflecting conserved roles in plant stress responses. Clustering by subcellular localization suggests evolutionary specialization, with GPXs adapting to specific organelle functions to manage oxidative stress effectively (**Figure 8**; Margis et al., 2008; Martins Alves et al., 2020). In the cytosol, they prevent oxidative damage and ensure metabolic and translational competence. In chloroplasts, GPXs safeguard photosynthetic components and minimize infection-related damage. Mitochondrial GPXs protect against dysfunction, maintaining cellular respiration and delaying PCD. Similarly, GPXs in the endoplasmic reticulum aid in protein folding and stress regulation, while apoplastic GPXs modulate extracellular ROS to activate defense signaling and systemic acquired resistance (SAR) (**Figure 9**) (Dietz and Hell, 2015; Tripathy and Oelmüller, 2012).

GPXs are integral to redox regulation and pathogen defense, acting across cellular compartments to balance ROS detoxification and signaling. Their evolutionary adaptations underline their versatility and importance in plant immunity. Further studies exploring sequence variations and functional roles could illuminate their contributions to stress resilience and crop improvement.

### 4.3 Multifaceted roles of GPXs in plant biological processes

The direct association of GPX2, GPX3, GPX4, GPX5, GPX7, and GPX8 with glutathione metabolic processes (**Figure 5**) suggests that the proteins can use glutathione as an electron donor in reducing H_2_O_2_ or organic and lipid hydroperoxides to maintain plant cell redox homeostasis (**Supplementary Table S2**) (Yang et al., 2005). However, plant GPXs are classified in the pyridoxine protein group due to an efficiently reduction of peroxide via the thioredoxin regenerating system (Iqbal et al., 2006; Margis et al., 2008). GPX1 and GPX6 participate in cellular processes responding to ROS, not only maintaining ROS levels’ homeostasis but also detecting and signaling ROS availability and redox imbalance (**Supplementary Table S2**) (Noctor et al., 2018).

Moreover, GPX3 plays a pivotal role in ABA-mediated stress signaling by interacting with phosphatases ABI1 and ABI2, key regulators of this pathway (**Figure 5**). It suppresses these phosphatases, acting as both a sensor and transducer of H_2_O_2_ signals during stress conditions (Miao et al., 2006; Paiva et al., 2021). While research linking GPX and ABA is limited, studies on rice GPX3 suggest it influences ABA signaling through H_2_O_2_-dependent and -independent mechanisms. In the H_2_O_2_-dependent pathway, GPX3 induction by ABA reduces oxidative stress by scavenging ROS, delaying senescence, and potentially enhancing resistance to biotrophic pathogens (Paiva et al., 2021). Conversely, this delay may increase susceptibility to necrotrophic pathogens (Apel and Hirt, 2004; Barna, 2022). In the H_2_O_2_-independent pathway, GPX3 modulates epigenetic responses by repressing S-glutathionylation of a signal transducer linked to histone modification (Jasmine and Joyous, 2022). It also influences the accumulation of ABA-responsive proteins, including histones, ubiquitin, actin, and vesicle-related proteins, which regulate gene expression and stress responses (Paiva et al., 2021). GPX3 further enhances ABA signaling by suppressing PP2C, a negative ABA regulator, and promoting genes like *OsDREB2A*, *OsABI5*, and *OsABA8ox3* (Paiva et al., 2021). This regulation impacts stomatal function, with GPX3-mediated ABA signaling inducing stomatal closure to prevent pathogen entry while potentially increasing susceptibility post-invasion by weakening cell wall defenses (Melotto et al., 2006; de Torres-Zabala et al., 2007; Ton et al., 2009; Lim et al., 2015; Hsu et al., 2021). Additionally, GPX3 may contribute indirectly to defense mechanisms through links to heat shock proteins (HSPs). HSP transcription factors activate genes for both HSPs and antioxidant enzymes under oxidative stress triggered by pathogens, underscoring GPX3’s multifaceted role in plant stress adaptation (Yurina, 2023) (**Figure 9**).

GPX1, GPX2, GPX3, GPX4, GPX5, GPX6, GPX7, and GPX8 associate with chloroplastic glutathione reductase proteins and mitochondrial glutathione transferases (DHAR2) related to the glutathione metabolic process (**Figure 5**). Regulating glutathione levels protects host plants against various pathogens. This is supported by the negative regulation of glutathione and susceptibility of tomato plants to *B. cinereae* (Kuźniak and Skłodowska, 2005), and in tomato plants infected with *P. syringae*, where glutathione levels decreased in susceptible cultivars but not in resistant ones (Kuźniak and SkŁodowska, 2004). GPXs and DHAR2 may cooperate in recycling glutathione and ascorbate during plant detoxification processes (Bobrovskikh et al., 2020) (**Figure 9**).

### 4.4 Comprehensive view of GPXs’ roles in plant stress responses and disease management

This SR highlights the pivotal role of GPXs in modulating plant resistance or susceptibility to pathogens. Key findings include, as first element, a differential GPX expression in resistant *vs*. susceptible plant varieties as observed in *Triticum aestivum* infected by barley yellow dwarf virus (BYDV). Resistant plants exhibited significantly higher GPX transcript levels and reduced H_2_O_2_ concentrations (4–5 times lower) compared to susceptible varieties. This reflects efficient ROS scavenging as a resistance mechanism (Wang et al., 2018). The second key finding concerns plant resistance increased by GPX mutations. In *A. thaliana*, loss-of-function mutations in GPX1 and GPX7 enhanced basal resistance to *P. syringae*. This resistance was linked to ROS- and salicylic acid (SA)-mediated signaling, which are critical for activating HR that confine pathogens to the infection site (Chang et al., 2009). And finally, the increase of susceptibility through GPX downregulation. In *C. sativa*, *Alternaria cucumerina* infection downregulated GPX7, causing ROS imbalances and enabling successful fungal colonization in susceptible plants (Sa et al., 2020). Proteomic studies corroborate these findings, showing that plants accumulate GPX proteins differently depending on the pathogen’s lifestyle. Interestingly, the consequences of the ROS-induced PCD depend on the context: in some cases, it facilitates pathogen colonization, increasing susceptibility, while in others, it restricts infection, halting pathogen progression and symptom development (Van Breusegem and Dat, 2006; Gadjev et al., 2008; Coll et al., 2011; Pinto et al., 2012; Daneva et al., 2016). This variation in GPX regulation underpins specific defense responses, as summarized in **Figure 4B**.

#### 4.4.1 GPX and biotrophic pathogens-specific responses

In plants, pathogen infection, including by oomycetes, bacteria, and fungi, typically begins when these organisms penetrate plant tissue through natural openings, wounds, or specialized structures (e.g., haustoria in oomycetes and fungi), and then colonize the apoplast, the intercellular space. Biotrophic pathogens rely on the apoplast for nutrients extracted from living cells (Kemen and Jones, 2012; Mapuranga et al., 2022). In the case of *V. vinifera* and the oomycete *Plasmopara viticola*, a compatible interaction leads to increased cytoplasmic GPX (VvGPX) accumulation, triggered by ROS signaling, which activates antioxidant enzymes like GPX to maintain redox balance, ultimately supporting pathogen growth (Milli et al., 2012; Koledenkova et al., 2022). Conversely, in *P. vulgaris* infected by the fungal pathogen *U. appendiculatus*, a decrease in the accumulation of chloroplastic and cytoplasmic PHGPX proteins (PvPHGPXs) was observed, which allowed the pathogen to bypass plant defense signaling (Lee et al., 2009). Additionally, biotrophic pathogens can suppress PAMP-induced immunity (PTI) by secreting effectors that inhibit ROS production, weakening plant defense responses. For example, *P. syringae* strains produce effectors that suppress both PAMP- and chitin-induced ROS, aiding pathogen colonization (Jamir et al., 2004; Fujikawa et al., 2006; Guo et al., 2009; Gimenez-Ibanez et al., 2018).

#### 4.4.2 GPX and viruses-specific responses

In an incompatible interaction with a virus, pathogens release viral elicitors that trigger a cascade of signaling pathways, activating plant defense mechanisms. These pathways include an oxidative burst, ROS generation, HR, pathogenesis-related protein activation, and SAR (Mandadi and Scholthof, 2013). However, ROS levels must remain transient to avoid excessive tissue damage, with antioxidant enzymes like GPXs maintaining redox balance (Gill and Tuteja, 2010; Hernández et al., 2016). In *V. unguiculata* infected by Cowpea severe mosaic virus (CPSMV), an increase in chloroplastic PHGPX proteins was observed at 6 dpi, while APX protein levels were low. The higher PHGPX abundance, coupled with lower APX, led to increased H_2_O_2_, promoting HR and resistance to the virus (Varela et al., 2017). In a study comparing non-inoculated (CPU), susceptible (CPI), and induced-resistant (MCPI) cowpeas, MCPI plants exhibited increased SOD activity and reduced CAT activity, causing a transient rise in H_2_O_2_ compared to CPI. In contrast, CPI plants showed increased CAT activity, potentially facilitating CPSMV infection. The study also found that in MCPI, elevated GPOX and reduced APX activity contributed to H_2_O_2_ buildup, crucial for defense establishment. Further analysis showed that MCPI plants controlled excess H_2_O_2_ through SOD and peroxiredoxins but limited viral spread by reducing CAT and APX activity. In susceptible plants, increased CAT and APX activity helped modulate ROS levels, promoting viral replication. These findings highlight the role of antioxidant enzymes in determining disease progression (Souza et al., 2017; Varela et al., 2017; Noronha Souza et al., 2020).

#### 4.4.3 GPX and necrotrophic pathogens-specific responses

Necrotrophic pathogens survive and develop by obtaining nutrients from dead plant cells, inducing cell death in the infected tissues through toxins and enzymes that cause nutrient leakage (Mengiste, 2012; Cho, 2015). This process triggers oxidative stress, which contributes to susceptibility. For instance, in a study of the necrotrophic fungi *Rhynchosporium secalis* and *Pyrenophora teres* infecting barley, ROS production (HO_2_•/O_2_-•) was observed in both susceptible and resistant plants. However, in later stages, ROS accumulation was only seen in susceptible plants. Pre-treatment with antioxidants like SOD and Mn(III) desferal reduced fungal growth and phenolic browning in susceptible plants. Additionally, resistant barley exhibited a sixfold increase in SOD activity when challenged with *P. teres* (Able, 2003). High antioxidant enzyme levels, such as GPXs, help mitigate oxidative stress caused by necrotrophic pathogens, enhancing plant resistance. For example, cytoplasmic GPX accumulation was noted as an induced response during the incompatible interaction of *B. napus* with *A. brassicae* (Sharma et al., 2007).

#### 4.4.4 GPX and hemibiotrophic pathogens-specific responses

Hemibiotrophic pathogens, which alternate between biotrophic and necrotrophic phases during infection, present unique challenges in plant defense (Glazebrook, 2005). During the biotrophic phase, ROS can inhibit pathogen spread, while in the necrotrophic phase, ROS favor the pathogen by promoting tissue death, which the pathogen uses for nutrient acquisition. The plant’s antioxidant response plays a critical role in this dynamic. For example, infection of *Leptosphaeria maculans* in *B. napus* cotyledons triggered H_2_O_2_ accumulation, and exogenous elicitors activated antioxidant enzymes, leading to a significant increase in activity of APX, GPX, GR, and SOD (Jindřichová et al., 2011). Similarly, *M. oryzae* infection in *O. sativa* resulted in a differential increase in antioxidant proteins, including OsPHGPX, especially in the early stages of a compatible interaction. In these cases, plant susceptibility may be linked to the efficiency of the antioxidant system in controlling ROS to prevent excessive cell death and facilitate pathogen colonization (Távora et al., 2021).

A study comparing antioxidant enzyme activity in susceptible and resistant plants infected with *M. oryzae* showed that all inoculated plants had increased CAT, APX, GPX, GST, and POX activity compared to controls. However, the level of response varied between species. For example, corn exhibited a greater increase in CAT activity than rice, while rice showed a decrease in GPX after inoculation. Antioxidant enzyme induction occurred earlier in resistant plants, which likely contributes to more effective defense (Gupta et al., 2021). In contrast, in an incompatible interaction with *C. gloeosporioides*, *M. domestica* cv. Fuji showed a notable increase in GPX accumulation, highlighting that GPX plays a critical role in detoxifying stressors and varies in its activity depending on the pathogen involved (Rockenbach et al., 2015). This underscores the importance of GPX as a key component of the plant’s antioxidant defense system.

#### 4.4.5 Evolutionary and ecological implications, and integrated disease management

The regulation and accumulation of GPXs in plants vary depending on the type of pathogen, influencing resistance or susceptibility. Increased GPX expression may enhance resistance against necrotrophic pathogens but can also increase susceptibility to biotrophs and viruses. This dynamic reflects the plant’s adaptive response to pathogen pressure in its environment, shaping specialized resistance profiles. However, such specialization might limit adaptability to new biotic challenges, as seen in evolutionary studies linking GPX regulation to altered ROS signaling and pathogen interactions. These changes can drive ecological trade-offs, where plants with high pathogen-specific resistance may underperform in low-pressure environments or against diverse threats (Herbette et al., 2011; Eloy et al., 2015; Camejo et al., 2016; Gupta et al., 2021). Integrated disease management provides a holistic strategy to address these challenges (Atkinson and Urwin, 2012). By combining GPX regulation with cultural management, crop rotation, optimized planting schedules, biological control, and genetic improvement, plants can better navigate the complexities of pathogen pressure and abiotic stress. These approaches reduce pathogen development, disrupt pest-host cycles, and enhance plant resilience, promoting sustainable cultivation and environmental health. Collectively, they ensure more robust, efficient, and adaptive agricultural systems (Mittler, 2006; Peshin and Dhawan, 2009; Bubici et al., 2019; Li et al., 2021; Yao et al., 2023; Ferrucho et al., 2024).

## 5 Conclusions

The SR synthesized information on plant GPXs against biotic stress from 2003 to 2023, providing a better understanding of their role against pathogens and identifying unresolved issues in the literature. While there is substantial knowledge on plant GPXs, specific functions for different isoforms, especially related to biotic stress, remain unclear. For example, clarifying if selenium can directly induce GPX activity against biotic stress or if more specific isoforms mimic animal antiapoptotic GPXs remains crucial. Answering such question could lead to the development of strategies to control necrotrophic or hemibiotrophic pathogens during plant host infection. Plant PHGPXs may function against pathogen-induced cell death, akin to animal antiapoptotic PHGPXs, yet few plant GPXs are explored for this. The susceptibility or resistance associated with plant GPXs depends on the lifestyle of the pathogen, but it’s not a strict rule, as plants possess various other mechanisms and antioxidant proteins that can be regulated and/or bypassed during pathogen attacks. Boosting the antioxidant activity of GPXs provides enhanced protection against pathogens, and employing strategies with exogenous inducers such as nano-Se, SA, pyrimidine derivatives, ASM, copper, and silica nanoparticles can aid in bolstering the defense provided by these proteins. GPXs not only act as antioxidants but also participate in glutathione metabolism, inhibit phosphatases, and detect/transduce H_2_O_2_ signals via ABA. While plant GPXs associated with biotic stress maintain characteristic motifs, notable changes occur, highlighting evolutionary relationships among them. This review underscores existing gaps in understanding plant GPXs against biotic stress, encouraging further experimental studies for strategic and biotechnological utilization in agriculture.

## Data Availability

The datasets presented in this study can be found in the article/**Supplementary Material**.
