# Supplementary material for: The family of glutathione peroxidase proteins and their role against biotic stress in plants: a systematic review
Source: Front Plant Sci. 2025 Feb 20;16:1425880. doi: 10.3389/fpls.2025.1425880 (PMC11882536; doi:10.3389/fpls.2025.1425880)
Supplement: Supplementary file 1 [file Table1.docx]

| **Section and Topic** | **Item #** | **Checklist item** | **Location where item is reported** |
| --- | --- | --- | --- |
| **TITLE** | | |  |
| Title | 1 | The glutathione peroxidase family of proteins and their role against biotic stress in plants: a systematic review | Title page, Page 1 |
| **ABSTRACT** | | |  |
| Abstract | 2 | Glutatation peroxidases (GPXs) are widely studied because they have essential roles in the elimination of reactive oxygen species (ROS), catalyzing the reduction of hydrogen or lipid peroxides, to prevent cell damage. Despite this, knowledge of GPXs in plants still has many gaps to be filled. Therefore, we present the first systematic review (RS) with the objective of investigating the action of GPXs and their role in protecting against cell death in plants under biotic stress. To guide the RS and avoid any bias, a protocol was developed that included inclusion and exclusion criteria based on the PRISMA guidelines. Three databases (PubMed, Science Direct and Springer) were used to identify relevant studies for this research, resulting in the selection of a total of 28 articles related to the proposed objective. The results highlighted evolutionary relationships between the GPXs studied and the importance of GPXs in defense against biotic stress in plants. As GPXs may be associated with defense against cell death, and their functional characteristics are analogous to animal antiapoptotic GPX proteins. The dataset extracted from studies of gene expression and protein accumulation in plants under different biotic stresses revealed that GPXs can increase both resistance and susceptibility to the pathogen. As GPXs participate not only in antioxidant processes, but they can also function as H2O2 detectors and signal transducers for components in the ABA signaling pathway during stress situations, and in interaction with processes related to heat shock proteins. The systematization of this information brings light to knowledge about the different molecular strategies involving GPX isoforms depending on the pathogen involved in the interaction with plants and experiment with systematized elements for discussion and strategic control actions against necrotrophic or hemibiotrophic pathogens. | Abstract,  Page 1 |
| **INTRODUCTION** | | |  |
| Rationale | 3 | The systematization of data on GPXs is an alternative to clarify and highlight the importance of these enzymes in the survival and resistance of plants to biotic stresses. Furthermore, it opens avenues for future work in investigating new functions and validating hypotheses. | Introduction, Page 3 |
| Objectives | 4 | General:  To propose the first systematization of plant Glutathione peroxidases and the different biological, biochemical, molecular and cellular protection functions against biotic stress, provided by the action of these enzymes.  Specifics:  Investigate the antioxidant activity of GPXs in plants, associated with different biological, biochemical and molecular functions against biotic stress.  Understand whether plant GPX enzymes can have action against cell death. | Introduction, Page 3 |
| **METHODS** | | |  |
| Eligibility criteria | 5 | The types of studies selected were defined based on the inclusion and exclusion criteria.  The inclusion criteria implied works in English; primary works; articles aligned with the objectives of the systematic review.  The exclusion criteria were: articles that were not aligned with the objectives of the systematic review; review articles; technical reports; book chapter; simple or expanded summaries; theses and dissertations; animal GPX articles; articles with abiotic stress. | Methods,  Planning,  Page 4 |
| Information sources | 6 | Searches for studies were carried out in previously selected databases: PubMed, Science Direct and Springer. The results obtained were imported into BIBTEX and MEDLINE format compatible with the Start software. | Methods,  Execution,  Page 4 |
| Search strategy | 7 | Advanced searches were carried out in the selected databases, with a “search string” in which the words were in the title and/or abstract. The string “Glutathione peroxidase” AND biotic stress AND cell death AND plant was used for this search, with the boolean connector AND used in the string to group the keywords and main terms. | Methods,  Execution,  Page 4 |
| Selection process | 8 | The Start program was used to organize, obtain articles without duplicates and for automated selection based on reading titles, abstracts and keywords. Articles meeting the inclusion criteria were selected. | Methods,  Execution,  Page 4 |
| Data collection process | 9 | The questions developed to achieve the objectives of the systematic review were:  1- What are the types of GPXs in plants?  1.1- Can GPXs exert highly specific biological functions in plants? Which?  1.2- Where are GPXs located in plants?  2- What are the main methods used to determine GPX activity?  3- What is the mechanism of action of GPXs in plants?  3.1- Are GPXs involved in the interaction with other proteins in response to biotic stress?  3.2- Which proteins can plant GPXs interact with?  4- Does the regulation of GPX expression confer protection on plants from biotic stress?  5- What is the role of GPXs in plants as redox sensors?  6- Does regulating the expression of GPXs in transgenic plants help protect against oxidative stress in defense against pathogens?  7- Can GPX control programmed death in plant cells, as well as animal GPXs?  8- Does selenium bioavailability increase the antioxidant potential of selenoprotein GPXs and non-selenoprotein GPXs? | Methods,  Planning,  Page 4 |
| Data items | 10a | Authors and year; Key words; Type of biotic stress (disease); Symptoms; Pathogen; Plant species; Study location; Specific biological functions; Types of GPXs; Location of GPXs; Techniques in evaluating GPXs activity; Functions shared between animal and plant GPXs; Mechanism of action in the plant; Interaction with other proteins; GPXs expression profile (mRNA); Accumulation of GPX proteins (proteomics); Role of GPXs as redox sensors; GPXs in PCD regulation; Change in GPX activity in the presence of selenium - Type of selenium compound; Another GPX Activator. | Methods,  Summarization,  Page 5 |
|  | 10b | All items mentioned above. | N/A |
| Study risk of bias assessment | 11 | We followed the exclusion and inclusion criteria for selecting articles, in addition, the PICOS strategy was adopted to guide the questions and the review was guided by the PRISMA guidelines. | Methods,  Planning,  Page 4 |
| Effect measures | 12 | We did not apply meta-analyses in the study. | N/A |
| Synthesis methods | 13a | We did not apply meta-analyses in the study. | N/A |
|  | 13b | We did not apply meta-analyses in the study. | N/A |
|  | 13c | We did not apply meta-analyses in the study. | N/A |
|  | 13d | We did not apply meta-analyses in the study. | N/A |
|  | 13e | We did not apply meta-analyses in the study. | N/A |
|  | 13f | We did not apply meta-analyses in the study. | N/A |
| Reporting bias assessment | 14 | The review was guided by the criteria pre-established in the execution protocol. | Methods,  Planning,  Page 4 |
| Certainty assessment | 15 | No additional methods were used in the analyzes | N/A |
| **RESULTS** | | |  |
| Study selection | 16a | A total of 872 articles were obtained from the search string in the Pubmed (32.8%), Science direct (56.5%) and Springer (9.3%) databases. In the StArt program, 5 duplicate articles were detected and 805 studies were excluded based on reading titles, abstracts and keywords, as they were not studies addressing plant GPXs associated with biotic stress. A total of 62 articles were read in full and underwent a second selection, still based on the inclusion and exclusion criteria, and of these, 34 articles met the exclusion criteria. Based on the selections made, 28 studies were considered eligible for this systematic review and were aligned with the objective of the study. | Results,  Page 6 |
|  | 16b | All excluded studies were outside the inclusion criteria. | N/A |
| Study characteristics | 17 | Data extraction from all selected articles was guided by the research questions. | Results,  Page 6-13 |
| Risk of bias in studies | 18 | All selected articles met the inclusion criteria. | Results,  Page 6 |
| Results of individual studies | 19 | All selected studies met the inclusion criteria. | Results,  Page 6-13 |
| Results of syntheses | 20a | We did not adopt meta-analysis in this research. | N/A |
|  | 20b | We did not adopt meta-analysis in this research. | N/A |
|  | 20c | We did not adopt meta-analysis in this research. | N/A |
|  | 20d | We did not adopt meta-analysis in this research. | N/A |
| Reporting biases | 21 | We do not apply risk analysis. | N/A |
| Certainty of evidence | 22 | We did not adopt meta-analysis in this research. | N/A |
| **DISCUSSION** | | |  |
| Discussion | 23a | - Plant GPXs protect cells against cell death, just like animal GPX4s or PHGPX; - The induction of GPXs assists in plant susceptibility or resistance and depends on the way of life of the interacting pathogen: The increase in the function of GPXs in cells increases detoxification, which is favorable to the survival of the biotrophic pathogen in the plant, but unfavorable to the necrotrophic pathogen. This can then increase susceptibility or resistance along with other plant functions. - Plant GPXs interact with biological processes: GPXs proteins may be involved not only in the cell's antioxidant activities, but also in sensing, signaling, signal transduction and participating in the glutathione metabolic process. Functions that may play a role in plant defense, such as the ABA signaling pathway during the stress response. - GPXs involved in biotic stress converge with each other: The formation of clades between the proteins in the present study, which had the same subcellular location, demonstrates a greater relationship between them, as in addition to having the same origin, they can act in the same location. | Discussion,  Page 13-20 |
|  | 23b | Although GPXs are important for plant defense against pathogens, there are still few studies demonstrating the functions of these proteins against biotic stress. | Discussion,  Page 14 |
|  | 23c | Few studies carry out the action of GPXs against biotic stress. | Discussion,  Page 14 |
|  | 23d | This RS sheds light on the gaps that still exist regarding plant GPXs against biotic stress, which can be remedied through new experimental studies that enable a more strategic and biotechnological use of this family of proteins in the control of microorganisms that directly affect agriculture. | Conclusion,  Page 21 |
| **OTHER INFORMATION** | | |  |
| Registration and protocol | 24a | The assessment was not recorded | N/A |
|  | 24b | The protocol is attached to be accessed. | Attachment 1 |
|  | 24c | There were no changes to the information provided. | N/A |
| Support | 25 | There was no funding foundation to carry out the review. | N/A |
| Competing interests | 26 | The authors declare that they have no known competing financial interests or personal relationships that could have influenced the work reported in this review. | N/A |
| Availability of data, code and other materials | 27 | The protocol, as well as the PRISMA form, questions that guided the review and the PICOS strategy will be in the article as tables or annexes to be consulted and verified. | Methods,  Planning,  Execution,  Summarization,  Page 4-6 |

*From:*  Page MJ, McKenzie JE, Bossuyt PM, Boutron I, Hoffmann TC, Mulrow CD, et al. The PRISMA 2020 statement: an updated guideline for reporting systematic reviews. BMJ 2021;372:n71. doi: 10.1136/bmj.n71

For more information, visit: <http://www.prisma-statement.org/>
