# Supplementary material for: The family of glutathione peroxidase proteins and their role against biotic stress in plants: a systematic review
Source: Front Plant Sci. 2025 Feb 20;16:1425880. doi: 10.3389/fpls.2025.1425880 (PMC11882536; doi:10.3389/fpls.2025.1425880)
Supplement: Supplementary file 2 [file Table2.docx]

| **Title** | The glutathione peroxidase family of proteins and their role against biotic stress in plants: a systematic review |
| --- | --- |
| **Researchers** | Maria Luíza do Carmo Santos;  Ariana Silva Santos;  Diogo Pereira Silva de Novais;  Natasha dos Santos Lopes  Carlos Priminho Pirovani;  Fabienne Micheli |
| **Description** | Glutathione peroxidase (GPX) is a family of proteins that have several isoforms with the potential to catalyze hydrogen peroxide (H2O2) or organic and lipid hydroperoxides in water or alcohols, respectively. To do this, these enzymes use glutathione (GSH) or thioredoxin (Trx) as electron donors. Furthermore, they may be involved in the regulation of cellular redox homeostasis by maintaining the thiol/disulfide or NADPH/NADP+ balance. GPXs are well known for their antioxidant role and are present in different animal and plant organisms.  Generally, proteins in this family are characterized as selenoproteins, as they often have the selenocysteine amino acid residue encoded by the UGA codon in their catalytic sites. The use of thioredoxin (Tr) or glutathione (GSH) as main electron donors appears to be associated with the presence of cysteine or selenocysteine in their catalytic site, which are often also reported to be selenium-independent or selenium-dependent.  GPXs proteins have three conserved motifs, which, although little detailed regarding their function, are present in most eukaryotic organisms. In these three motifs there are three main catalytic centers presented as cysteine(Cys) or selenocysteine(SeCys)-Glutamine(Gln)-Tryptophan(Trp).  In plants, these proteins vary in location, between chloroplasts, mitochondria, cytoplasm or can be secreted. Furthermore, plant GPXs more frequently have cysteine than selenocysteine in their catalytic site, which for some authors justifies a lower activity compared to animal proteins. But there is still controversy on this issue because a protein with high peroxidase activity in plants has already been found, and even though this activity has less effect when compared to animal protein, it seems to be sufficient to protect cells from oxidative damage in plants.  The systematization of previous information about GPXs is an alternative to clarify and highlight the importance of these enzymes in the survival and resistance of plants to biotic stress. Furthermore, it opens new avenues for future work in investigating new functions and validating hypotheses. |
| **General objective** | Propose the first systematization of plant Glutathione peroxidases and the different biological, biochemical, molecular and cellular protection functions against biotic stress, provided by the action of these enzymes. |
| **Specific objectives** | Investigate the antioxidant activity of GPXs in plants, associated with different biological, biochemical and molecular functions against biotic stress.  Understand whether plant GPX enzymes can act against cell death. |
| **Questions** | 1- What are the types of GPXs in plants?  1.2- Can GPXs exert highly specific biological functions in plants? Which?  1.3- Where are GPXs located in plants?  2- What are the main methods used to determine GPX activity?  3- What is the mechanism of action of GPXs in plants?  3.1- Are GPXs involved in the interaction with other proteins in response to biotic stress?  3.2- Which proteins can plant GPXs interact with?  4- Does the regulation of GPX expression confer protection on plants from biotic stress?  5- What is the role of GPXs in plants as redox sensors?  6- Does regulating the expression of GPXs in transgenic plants help protect against oxidative stress in defense against pathogens?  7- Can GPX control programmed death in plant cells, as well as animal GPXs?  8- Does selenium bioavailability increase the antioxidant potential of selenoprotein GPXs and non-selenoprotein GPXs? |
| **Key words** | Antioxidant enzyme, stress tolerance, biotic stress |
| **Search string** | *“Glutathione peroxidase” and biotic stress and cell death and plant* |
| **Search source selection criteria** | Scientific articles indexed in peer review journals;  Databases that offer greater options according to advanced search criteria. |
| **Research method** | Advanced searches, in selected databases, with “search string” in which the words were in the title and/or summary;  Use of programs (Start) to organize and obtain articles without duplicates;  PICOS analyses. |
| **Database for research** | PubMed; Springer; Science Direct |
| **Inclusion criteria** | - Works in English; - Primary works; - Articles that are aligned with the objectives of the systematic review |
| **Exclusion Criteria** | - Articles that are not aligned with the objectives of the systematic review; - Review articles; - Technical reports; - Book chapter; - Simple or expanded summaries; - Theses and dissertations; - Articles on animal GPXs; - Abiotic stress |
| **Definition of types of studies** | Based on inclusion and exclusion criteria |
| **Initial selection of studies** | - Title; - Summary; - Key words. |
| **Final selection of studies** | - Present the inclusion criteria; - Not presenting one of the exclusion criteria. |
| **Data Extraction Strategy** | - Full paper (methodology + results + discussion + conclusion); - Key words; - Species of the plants studied; - Study location; - Specific biological functions; - Types of GPXs; - Techniques used to evaluate GPX activity; - Functions shared between GPXs of animal and plant species; - Mechanism of action of GPXs; - GPXs interaction with other proteins; - GPX expression profile; - Action of GPXs in defense against pathogens; - Response against which pathogen; - The role of GPXs as redox sensors; - GPXs in the control of cell death; - Change in GPX activity in the presence of selenium; - Type of selenium compound and concentration, associated with GPX activity; - Authors and year of the article. |
| **Data summarization** | Graphs, tables and figures. |
