# Supplementary material for: The family of glutathione peroxidase proteins and their role against biotic stress in plants: a systematic review
Source: Front Plant Sci. 2025 Feb 20;16:1425880. doi: 10.3389/fpls.2025.1425880 (PMC11882536; doi:10.3389/fpls.2025.1425880)
Supplement: Supplementary file 3 [file Table3.docx]

**Supplementary materials**

**Table S1.** Inclusion and exclusion criteria used in selecting studies for the SR.

| **Inclusion criteria** | **Exclusion criteria** |
| --- | --- |
| Scientific articles in English | Scientific articles that are not aligned with the objectives of the systematic review |
| Primary scientific articles | Review articles |
| Scientific articles that are aligned with the objectives of the systematic review | Technical reports |
|  | Book chapters |
|  | Simple or expanded summaries |
|  | Theses and dissertations |
|  | Animal GPX articles |
|  | Articles with abiotic stress |

**Table S2.** Identity between the proteins extracted from the selected studies and their orthologs/homologs in *Arabidopsis thaliana*, obtained from the STRING server.

| **Proteins from selected studies** | | | ***Arabidopsis thaliana* sequences** | | **Identity (%)** | **Bitscore** |
| --- | --- | --- | --- | --- | --- | --- |
| **ID** | **Name** | **Species** | **ID** | **Name** |  |  |
| XP_004145493.1 | CsGPX7 | *Citrus sinensis* | AT2G25080 | GPX1 | 75.0 | 324.7 |
| AAQ03092.1 154 | MdGPX | *Malus domestica* | AT4G11600 | GPX6 | 88.6 | 308.1 |
| XP_044968631.1 | TaPHGPX | *Triticum aestivum* | AT4G11600 | GPX6 | 71.0 | 255.4 |
| ACV52584.1 | NbPHGPX | *Nicotiana benthamiana* | AT4G11600 | GPX6 | 83.3 | 162.9 |
| XP_002272606.1 | VvPHGPX | *Vitis vinifera* | AT4G11600 | GPX6 | 83.3 | 298.9 |
| A0A0N7KJH5 | OsPHGPX | *Oryza sativa* | AT4G11600 | GPX6 | 86.6 | 212.6 |
| NP_001233800.2 | SlGPX | *Solanum lycopersicum* | AT2G31570 | GPX2 | 76.6 | 281.2 |
| ACG28364.1 | ZmPHGPX | *Zea mays* | AT4G11600 | GPX6 | 83.7 | 295.8 |
| NP_001289784.1 | NnPHGPX | *Nelumbo nucifera* | AT4G11600 | GPX6 | 79.5 | 291.6 |
| AAC78466.1 | ZaPHGPX | *Zantedeschia aethiopica* | AT2G25080 | GPX1 | 74.9 | 313.5 |
| NP_194915.2 | AtGPX7 | *Arabidopsis thaliana* | AT4G31870 | GPX7 | 100.0 | 469.5 |
| NP_180080.1 | AtGPX1 | *Arabidopsis thaliana* | AT2G25080 | GPX1 | 100.0 | 469.5 |
| NP_180715.1 | AtGPX2 | *Arabidopsis thaliana* | AT2G31570 | GPX2 | 100.0 | 349.0 |
| NP_001189742.1 | AtGPX3 | *Arabidopsis thaliana* | AT2G43350 | GPX3 | 100.0 | 420.2 |
| NP_564813.1 | AtGPX8 | *Arabidopsis thaliana* | AT1G63460 | GPX8 | 100.0 | 350.1 |
| NP_192897.2 | AtGPX6 | *Arabidopsis thaliana* | AT4G11600 | GPX6 | 100.0 | 439.9 |
| NP_191867.1 | AtGPX5 | *Arabidopsis thaliana* | AT3G63080 | GPX5 | 100.0 | 344.4 |
| NP_566128.1 | AtGPX4 | *Arabidopsis thaliana* | AT2G48150 | GPX4 | 100.0 | 346.7 |
| ANA75381.1 | HbGPX | *Hevea brasiliensis* | AT2G31570 | GPX2 | 78.4 | 283.5 |
| PGSC0003DMG402004978 | StGPX | *Solanum tuberosum* | AT1G63460 | GPX8 | 73.6 | 266.5 |
| Q06652 | CsPHGPX | *Citrus sinensis* | AT4G11600 | GPX6 | 82.8 | 285.4 |
| O23968 | HaGPX2 | [*Helianthus annuus*](https://www.ncbi.nlm.nih.gov/Taxonomy/Browser/wwwtax.cgi?mode=Info&id=4232) | AT3G63080 | GPX5 | 73.4 | 261.9 |
| CAA04142.1 | PvPHGPX | *Phaseolus vulgaris* | AT2G25080 | GPX1 | 57.2 | 127.9 |
| ABE92133.1 | PcPHGPX | *Phaseolus coccineus* | AT2G25080 | GPX1 | 57.2 | 127.9 |
| XP_007011699.1 | TcPHGPX | *Theobroma cacao* | AT2G25080 | GPX1 | 78.3 | 344.7 |
| XP_007026518.1 | TcGPX2 | *Theobroma cacao* | AT2G43350 | GPX3 | 68.9 | 265.0 |
| XP_007050669.1 | TcGPX4 | *Theobroma cacao* | AT3G63080 | GPX5 | 73.4 | 261.2 |
| XP_007040204.1 | TcGPX6 | *Theobroma cacao* | AT4G11600 | GPX6 | 83.5 | 304.3 |
| XP_007040205.1 | TcGPX8 | *Theobroma cacao* | AT1G63460 | GPX8 | 69.9 | 269.2 |
| XP_015059468.1 | SpGPX8 | *Solanum pennellii* | AT1G63460 | GPX8 | 88.6% | 308.1 |
| NP_001149631.1 | ZmGPX | *Zea mays* | AT4G11600 | GPX6 | 83.7% | 295.8 |

**Table S3.** Studies that used, among others, the GPX enzymatic activity as evaluation method of the plant response to infection by pathogens.

| **GPX activity** | **Type of biotic stress** | **Tolerance or resistance** | **Inducer** | | **Method used to measure GPX activity** | **Ref.** |
| --- | --- | --- | --- | --- | --- | --- |
|  |  |  | **Presence** | **Type** |  |  |
| Decreased | Hypersensitive cell death | Increased | No | _ | Peroxide reduction measured from the NADPH oxidation rate | [1] |
| Increased | Spot disease | Increased | Yes | Pyrimidine derivative (2-amino 4-6-dimethyl pyridine) (DPD) | Peroxide reduction measured from the NADPH oxidation rate | [2] |
| Increased | Bacterial canker | Increased | Yes | Acibenzolar-S-methyl (benzo [1,2,3] thiadiazole-7-carbothioic acid S-methyl ester) (ASM) | Peroxide reduction measured from the NADPH oxidation rate | [3] |
| Increased | Bacterial spot disease | No increase | No | _ | Peroxide reduction measured from the NADPH oxidation rate | [4] |
| Increased | Root knot | No increase | No | _ | Activity measured by absorbance at 420 nm | [5] |
| Increased | Yellow Mosaic Disease (YMD) or “Yellow plague of kharif pulses” | No increase | No | _ | Peroxide reduction measured from the NADPH oxidation rate | [6] |
| Increased | Marginal necrosis in the leaves | Increased | Yes | Copper and potassium silicate nanoparticles | The absorbance measured at a wavelength of 412 nm | [7] |
| Increased | Yellow spot disease | Increased | Yes | Salicylic acid (SA) | Activity detected using assay kit (Nanjing Jiancheng Institute of Biological Engineering) | [8] |
| Increased | Vascular wilt disease and crown and root rot | No increase | Yes | Carbon nanotubes  Graphene nanoplatelets | Absorbance measured at a wavelength of 412 nm | [9] |
| Increased | Sclerotinia stem rot | Increased | No | _ | Peroxide reduction measured from the NADPH oxidation rate | [10] |
| Increased | Chlorosis and cell death | Increased | Yes | Nano-selenium | Commercial assay kits (Nanjing Jiancheng Institute of Bioengineering) | [11] |

**Table S4.** Studies selected in this SR, according to the inclusion and exclusion criteria (see Table 1 and Table S1).

| **Title** | **DOI** | **Ref.** |
| --- | --- | --- |
| Arabidopsis Chloroplastic Glutathione Peroxidases Play a Role in Cross Talk between Photooxidative Stress and Immune Responses | 10.1104/pp.109.135566 | [1] |
| Early oxidative burst and anthocyanin-mediated antioxidant defense mechanism impart resistance against *Sclerotinia sclerotiorum* in Indian mustard | 10.1016/j.pmpp.2022.101847 | [10] |
| Effect of carbon-based nanomaterials on *Fusarium* wilt in tomato | 10.1016/j.scienta.2021.110586 | [9] |
| Efficacy of a pyrimidine derivative to control spot disease on Solanum melongena caused by Alternaria alternata | 10.1016/j.jare.2012.07.008 | [2] |
| Expression analysis of ROS producing and scavenging enzyme-encoding genes in rubber tree infected by Pseudocercospora ulei | 10.1016/j.plaphy.2016.03.022 | [12] |
| Expression of biotic stress response genes to Phytophthora infestans inoculation in White Lady, a potato cultivar with race-specific resistance to late blight | 10.13140/RG.2.1.2748.3283 | [13] |
| Gel-free/label-free proteomic, photosynthetic, and biochemical analysis of cowpea (*Vigna unguiculata* [L.] Walp.) resistance against *Cowpea severe mosaic virus* (CPSMV) | 10.1016/j.jprot.2017.05.003 | [14] |
| Gene architecture and expression analyses provide insights into the role of glutathione peroxidases (GPXs) in bread wheat (Triticum aestivum L.) | 10.1016/j.jplph.2018.02.006 | [15] |
| Histological and proteomics analysis of apple defense responses to the development of *Colletotrichum gloeosporioides* on leaves | 10.1016/j.pmpp.2015.01.003 | [16] |
| Induction of disease resistance by the plant activator, acibenzolar-S- methyl (ASM), against bacterial canker (Clavibacter michiganensis subsp. michiganensis) in tomato seedlings | 10.1016/S0168-9452(03)00302-9 | [3] |
| Investigation of the mechanism of adult-stage resistance to barley yellow dwarf virus associated with a wheat–Thinopyrum intermedium translocation | 10.1016/j.cj.2018.02.002 | [17] |
| iTRAQ-based quantitative proteomics reveals a ferroptosis-like programmed cell death in plants infected by a highly virulent tobacco mosaic virus mutant 24A+UPD | 10.1186/s42483-019-0043-5 | [18] |
| Nano-selenium promotes the product quality and plant defense of *Salvia miltiorrhiza* by inducing tanshinones and salvianolic acids accumulation | 10.1016/j.indcrop.2023.116436 | [11] |
| Physiological changes in tomato leaves arising from *Xanthomonas gardneri* infection | 10.1016/j.pmpp.2015.10.001 | [4] |
| Polyamines induced nematode stress tolerance in *Solanum lycopersicum* through altered physico-chemical attributes | 10.1016/j.pmpp.2020.101544 | [5] |
| Proteome-level changes in two *Brassica napus* lines exhibiting differential responses to the fungal pathogen *Alternaria brassicae* | j.plantsci.2006.07.016 | [19] |
| Proteomic analysis of the compatible interaction between *Vitis vinifera* and *Plasmopara viticola* | j.jprot.2011.11.006 | [20] |
| Quantitative Proteomic Analysis of Bean Plants Infected by a Virulent and Avirulent Obligate Rust Fungus | mcp.M800156-MCP200 | [21] |
| Role of glutathione in methylglyoxal detoxification pathway during yellow mosaic virus (YMV) infection in black gram (Vigna mungo (L.) Hepper) | 10.1016/j.pmpp.2020.101513 | [6] |
| Salicylic acid enhances the resistance of *Neoporphyra haitanensis* to pathogen *Vibrio mediterranei* 117-T6 | 10.1016/j.aquaculture.2022.738773 | [8] |
| Salicylic acid seed priming instigates defense mechanism by inducing PR-proteins in Solanum melongena L. upon infection with Verticillium dahliae Kleb | 10.1016/j.plaphy.2017.05.012 | [22] |
| Shotgun proteomics coupled to transient-inducible gene silencing reveal rice susceptibility genes as new sources for blast disease resistance | 10.1016/j.jprot.2021.104223 | [23] |
| The application of copper nanoparticles and potassium silicate stimulate the T tolerance to *Clavibacter michiganensis* in tomato plants | 10.1016/j.scienta.2018.10.007 | [7] |
| The glutathione peroxidase family of Theobroma cacao: Involvement in the oxidative stress during witches' broom disease | 10.1016/j.ijbiomac.2020.08.222 | [24] |
| Tomato Phospholipid Hydroperoxide Glutathione Peroxidase Inhibits Cell Death Induced by Bax and Oxidative Stresses in Yeast and Plants | [10.1104/pp.103.038091](https://doi.org/10.1104/pp.103.038091) | [25] |
| Transcriptional profiling of canola (*Brassica napus* L.) responses to the fungal pathogen *Sclerotinia sclerotiorum* | 10.1016/j.plantsci.2007.04.012 | [26] |
| Transcriptome analysis of mechanisms and candidate genes associated with cucumber response to cucumber alternaria leaf spot infection | 10.1016/j.pmpp.2020.101490 | [27] |
| Transcriptome-wide identification and transcriptional profiling reveal remarkable expression modulation of redox genes in *Zingiber zerumbet* against *Pythium myriotylum* | 10.1016/j.pmpp.2022.101885 | [28] |

**Table S5.** GPX proteins used for alignment analysis and Neighbor-joining tree construction in ClustalW. nd: non-determined. (*) The studies gene sequences were used to obtain the corresponding protein sequences. (**) Homologous proteins according to the sequence provided by the study. SGN: SOL Genomics Network.

| **Plant species** | **GPX name** | **ID** | **Homologous plant specie** | **Database** | **References** |
| --- | --- | --- | --- | --- | --- |
| *Cucumis sativus* L. | CsGPX7 | [XP_004145493.1*](https://www.ncbi.nlm.nih.gov/protein/XP_004145493.1?report=genbank&log$=prottop&blast_rank=1&RID=EA51HYGT01R) | _ | NCBI | [27] |
| *Malus domestica* | MdGPX | AAQ03092.1 154 | _ | NCBI | [16] |
| *Triticum aestivum* L. | TaPHGPX | XP_044968631.1* | _ | NCBI | [17] |
| *Nicotiana benthamiana* | NbPHGPX | ACV52584.1* | _ | NCBI | [18] |
| *Brassica napus* | GPX (MdGPX) | AAQ03092.1 ** | *Malus domestica* | NCBI | [19] |
| *Vitis vinifera* | VvPHGPX | XP_002272606.1* | _ | NCBI | [20] |
| *Solanum melongena* L. | SpGPX8 | XP_015059468.1* | _ | NCBI | [22] |
| *Oryza sativa* L. | OsPHGPX | A0A0N7KJH5 | _ | Uniprot | [23] |
| *Lycopersicon esculentum* | SlGPX | NP_001233800.2** | *Solanum lycopersicum* | NCBI | [25] |
| *Zingiber zerumbet* | ZmPHGPX | ACG28364.1** | *Zea mays* | NCBI | [28] |
| *Zingiber zerumbet* | NnPHGPX | NP_001289784.1** | *Nelumbo nucifera* | NCBI |  |
| *Zingiber zerumbet* | ZaGPX | AAC78466.1** | *Zantedeschia aethiopica* | NCBI |  |
| *Zingiber zerumbet* | ZmGPX | NP_001149631.1** | *Zea mays* | NCBI |  |
| *Arabidopsis thaliana* | AtGPX7 | NP_194915.2 | _ | NCBI | [1] |
| *Arabidopsis thaliana* | AtGPX1 | NP_180080.1 | _ | NCBI |  |
| *Arabidopsis thaliana* | AtGPX2 | NP_180715.1 | _ | NCBI |  |
| *Arabidopsis thaliana* | AtGPX3 | NP_001189742.1 | _ | NCBI |  |
| *Arabidopsis thaliana* | AtGPX8 | NP_564813.1 | _ | NCBI |  |
| *Arabidopsis thaliana* | AtGPX6 | NP_192897.2 | _ | NCBI |  |
| *Arabidopsis thaliana* | AtGPX5 | NP_191867.1 | _ | NCBI |  |
| *Arabidopsis thaliana* | AtGPX4 | NP_566128.1 | _ | NCBI |  |
| *Hevea brasiliensis* | HbGPX | ANA75381.1* | _ | NCBI | [12] |
| *Solanum tuberosum* | StGPX | PGSC0003DMG402004978 | _ | SGN | [13] |
| *Vigna unguiculata* | CsPHGPX | Q06652** | *Citrus sinensis* | NCBI | [14] |
| *Vigna unguiculata* | HaGPX2 | O23968** | [*Helianthus annuus*](https://www.ncbi.nlm.nih.gov/Taxonomy/Browser/wwwtax.cgi?mode=Info&id=4232) | NCBI |  |
| *Phaseolus vulgaris* | PvPHGPX | CAA04142.1 | _ | NCBI | [21] |
| *Phaseolus vulgaris* | PcPHGPX | ABE92133.1** | *Phaseolus coccineus* | NCBI |  |
| *Theobroma cacao* | TcPHGPX | XP_007011699.1 | _ | NCBI | [24] |
| *Theobroma cacao* | TcGPX2 | XP_007026518.1 | _ | NCBI |  |
| *Theobroma cacao* | TcGPX4 | XP_007050669.1 | _ | NCBI |  |
| *Theobroma cacao* | TcGPX6 | XP_007040204.1 | _ | NCBI |  |
| *Theobroma cacao* | TcGPX8 | XP_007040205.1 | _ | NCBI |  |
| *Triticum aestivum* | TaGPX1 (A, A1, A2, B, D) | nd | nd | nd | [15] |
| *Triticum aestivum* | TaGPX2-B | nd | nd | nd |  |
| *Triticum aestivum* | TaGPX3 (A, B, U) | nd | nd | nd |  |
| *Triticum aestivum* | TaGPX4 (B, D, U) | nd | nd | nd |  |
| *Triticum aestivum* | TaGPX5 (A, A2, B1, B2, D) | nd | nd | nd |  |

**Figure S1.** Complete alignment obtained using GPX protein sequences from the analyzed studies. The alignment was carried out by uploading the sequences of amino acid residues corresponding to the studied GPXs into Clustal W.

**References**

[1] C.C.C. Chang, I. Slesak, L. Jordá, A. Sotnikov, M. Melzer, Z. Miszalski, P.M. Mullineaux, J.E. Parker, B. Karpinska, and S. Karpinski, Arabidopsis chloroplastic glutathione peroxidases play a role in cross talk between photooxidative stress and immune responses. Plant Physiol 150 (2009) 670-683.

[2] N.M. Hassan, M.I. Abu-Doubara, M.A. Waly, and M.M. Nemat Alla, Efficacy of a pyrimidine derivative to control spot disease on Solanum melongena caused by Alternaria alternata. Journal of advanced research 4 (2013) 393-401.

[3] S. Soylu, Ö. Baysal, and E.M. Soylu, Induction of disease resistance by the plant activator, acibenzolar-S-methyl (ASM), against bacterial canker (Clavibacter michiganensis subsp. michiganensis) in tomato seedlings. Plant Science 165 (2003) 1069-1075.

[4] P.R. Silveira, K.J.T. Nascimento, C.C.L. Andrade, W.M.S. Bispo, J.R. Oliveira, and F.A. Rodrigues, Physiological changes in tomato leaves arising from Xanthomonas gardneri infection. Physiological and Molecular Plant Pathology 92 (2015) 130-138.

[5] A. Khajuria, and P. Ohri, Polyamines induced nematode stress tolerance in Solanum lycopersicum through altered physico-chemical attributes. Physiological and Molecular Plant Pathology 112 (2020) 101544.

[6] Y.J. Singh, S.K. Grewal, and R.K. Gill, Role of glutathione in methylglyoxal detoxification pathway during yellow mosaic virus (YMV) infection in black gram (Vigna mungo (L.) Hepper). Physiological and Molecular Plant Pathology 111 (2020) 101513.

[7] C.F. Cumplido-Nájera, S. González-Morales, H. Ortega-Ortíz, G. Cadenas-Pliego, A. Benavides-Mendoza, and A. Juárez-Maldonado, The application of copper nanoparticles and potassium silicate stimulate the tolerance to Clavibacter michiganensis in tomato plants. Sci Hortic 245 (2019) 82-89.

[8] S. Zhu, Z. Jin, J. Chen, R. Yang, Q. Luo, T. Wang, P. Zhang, and H. Chen, Salicylic acid enhances the resistance of Neoporphyra haitanensis to pathogen Vibrio mediterranei 117-T6. Aquaculture 562 (2023) 738773.

[9] Y. González-García, G. Cadenas-Pliego, Á.G. Alpuche-Solís, R.I. Cabrera, and A. Juárez-Maldonado, Effect of carbon-based nanomaterials on Fusarium wilt in tomato. Sci Hortic 291 (2022).

[10] M. Singh, R. Avtar, N. Lakra, A. Pal, V.K. Singh, R. Punia, N. Kumar, M. Bishnoi, N. Kumari, R.S. Khedwal, and R.R. Choudhary, Early oxidative burst and anthocyanin-mediated antioxidant defense mechanism impart resistance against Sclerotinia sclerotiorum in Indian mustard. Physiol Mol Plant Pathol 120 (2022) 101847.

[11] Y. Zhang, T. Zhang, Y. Pan, L. Ma, Y. Fang, C. Pan, Y. Qiang, X. Cao, and H. Xu, Nano-selenium promotes the product quality and plant defense of Salvia miltiorrhiza by inducing tanshinones and salvianolic acids accumulation. Ind Crops Prod 195 (2023) 116436.

[12] D.M. Koop, M. Rio, X. Sabau, S.E. Almeida Cardoso, C. Cazevieille, J. Leclercq, and D. Garcia, Expression analysis of ROS producing and scavenging enzyme-encoding genes in rubber tree infected by Pseudocercospora ulei. Plant Physiol Biochem 104 (2016) 188-99.

[13] R. Hajianfar, B. Kolics, I. Cernák, I. Wolf, Z. Polgár, and J. Taller, Expression of biotic stress response genes to Phytophthora infestans inoculation in White Lady, a potato cultivar with race-specific resistance to late blight. Physiological and Molecular Plant Pathology 93 (2016) 22-28.

[14] A.L.N. Varela, S. Komatsu, X. Wang, R.G.G. Silva, P.F.N. Souza, A.K.M. Lobo, I.M. Vasconcelos, J.A.G. Silveira, and J.T.A. Oliveira, Gel-free/label-free proteomic, photosynthetic, and biochemical analysis of cowpea (Vigna unguiculata [L.] Walp.) resistance against Cowpea severe mosaic virus (CPSMV). J Proteomics 163 (2017) 76-91.

[15] S. Tyagi, Himani, J.K. Sembi, and S.K. Upadhyay, Gene architecture and expression analyses provide insights into the role of glutathione peroxidases (GPXs) in bread wheat (Triticum aestivum L.). J Plant Physiol 223 (2018) 19-31.

[16] M.F. Rockenbach, J.I. Boneti, G.C. Cangahuala-Inocente, M.C.A. Gavioli-Nascimento, and M.P. Guerra, Histological and proteomics analysis of apple defense responses to the development of Colletotrichum gloeosporioides on leaves. Physiological and Molecular Plant Pathology 89 (2015) 97-107.

[17] X. Wang, W. Rong, Y. Liu, X. Wang, and Z. Zhang, Investigation of the mechanism of adult-stage resistance to barley yellow dwarf virus associated with a wheat–Thinopyrum intermedium translocation. Crop J 6 (2018) 394-405.

[18] M. Macharia, P.P. Das, N.I. Naqvi, and S.-M. Wong, iTRAQ-based quantitative proteomics reveals a ferroptosis-like programmed cell death in plants infected by a highly virulent tobacco mosaic virus mutant 24A+UPD. Phytopathol Res 2 (2020) 1.

[19] N. Sharma, M.H. Rahman, S. Strelkov, M. Thiagarajah, V.K. Bansal, and N.N.V. Kav, Proteome-level changes in two Brassica napus lines exhibiting differential responses to the fungal pathogen Alternaria brassicae. Plant Science 172 (2007) 95-110.

[20] A. Milli, D. Cecconi, L. Bortesi, A. Persi, S. Rinalducci, A. Zamboni, G. Zoccatelli, A. Lovato, L. Zolla, and A. Polverari, Proteomic analysis of the compatible interaction between Vitis vinifera and Plasmopara viticola. J Proteomics 75 (2012) 1284-302.

[21] J. Lee, J. Feng, K.B. Campbell, B.E. Scheffler, W.M. Garrett, S. Thibivilliers, G. Stacey, D.Q. Naiman, M.L. Tucker, M.A. Pastor-Corrales, and B. Cooper, Quantitative proteomic analysis of bean plants infected by a virulent and avirulent obligate rust fungus. Mol Cell Proteomics 8 (2009) 19-31.

[22] H.M. Mahesh, M. Murali, M. Anup Chandra Pal, P. Melvin, and M.S. Sharada, Salicylic acid seed priming instigates defense mechanism by inducing PR-Proteins in Solanum melongena L. upon infection with Verticillium dahliae Kleb. Plant Physiol Biochem 117 (2017) 12-23.

[23] F. Távora, R. Bevitori, R.N. Mello, M. Cintra, O.B. Oliveira-Neto, W. Fontes, M.S. Castro, M.V. Sousa, O.L. Franco, and A. Mehta, Shotgun proteomics coupled to transient-inducible gene silencing reveal rice susceptibility genes as new sources for blast disease resistance. J Proteomics 241 (2021) 104223.

[24] A.M. Martins Alves, S. Pereira Menezes Reis, K. Peres Gramacho, and F. Micheli, The glutathione peroxidase family of Theobroma cacao: Involvement in the oxidative stress during witches' broom disease. Int J Biol Macromol 164 (2020) 3698-3708.

[25] S. Chen, Z. Vaghchhipawala, W. Li, H. Asard, and M.B. Dickman, Tomato phospholipid hydroperoxide glutathione peroxidase inhibits cell death induced by Bax and oxidative stresses in yeast and plants. Plant Physiol 135 (2004) 1630-1641.

[26] B. Yang, S. Srivastava, M.K. Deyholos, and N.N.V. Kav, Transcriptional profiling of canola (Brassica napus L.) responses to the fungal pathogen Sclerotinia sclerotiorum. Plant Science 173 (2007) 156-171.

[27] R. Sa, D. Liu, L. Chen, L. Liu, Y. Zhang, X. Zhang, and C. Xu, Transcriptome analysis of mechanisms and candidate genes associated with cucumber response to cucumber alternaria leaf spot infection. Physiological and Molecular Plant Pathology 111 (2020) 101490.

[28] T.E. Alex, V.S. Nath, L. Varghese, K.A. Geetha, L. Augustine, V.M. Ramaswamy, and G. Thomas, Transcriptome-wide identification and transcriptional profiling reveal remarkable expression modulation of redox genes in Zingiber zerumbet against Pythium myriotylum. Physiol Mol Plant Pathol 121 (2022) 101885.
